# Robust Antifogging and Antifouling Coating Tailored with Zwitterionic Nanocellulose for Multi‐Functional Applications

**DOI:** 10.1002/advs.202509204

**Published:** 2025-09-10

**Authors:** Yanyi Duan, Jiangjiexing Wu, Aihua Qiao, Zheng Zhang, Jiaxin Shang, Xiaohui Mao, Xiaohui Yan, Nan Wang, Peng Zhong, Xiaobo Li, Xavier Banquy, Wei Qi, Rongxin Su

**Affiliations:** ^1^ State Key Laboratory of Chemical Engineering Tianjin Key Laboratory of Membrane Science and Desalination Technology School of Chemical Engineering and Technology Tianjin University Tianjin 300072 P. R. China; ^2^ School of Marine Science and Technology Tianjin University Tianjin 300072 P. R. China; ^3^ Tianjin Stomatological Hospital School of Medicine Nankai University Tianjin 300071 P. R. China; ^4^ College of Materials Science and Engineering Donghua University 2999 North Renmin Road Shanghai 201620 P. R. China; ^5^ State Key Laboratory of Chinese Medicine Modernization Tianjin University of Traditional Chinese Medicine 10 Poyanghu Road Tianjin 301617 P. R. China; ^6^ Faculty of Pharmacy Université de Montréal Montréal QC H3T 1J4 Canada

**Keywords:** antifogging, antifouling, biocompatible, superhydrophilic, zwitterionic nanocellulose

## Abstract

Biofouling often occurs simultaneously with fogging, presenting significant challenges to visibility, safety, and operational efficiency. The development of biocompatible coatings that offer both antifouling performance and stability under fogging conditions is highly sought after. A method to form multifunctional coatings is presented, utilizing a zwitterionic nanocellulose composite material that demonstrates both antifogging and antifouling properties, suitable for application on various surfaces. Cellulose nanofibers are modified with lysine to impart zwitterionic characteristics, enhancing hydrophilicity and antifouling performance. Polyacrylic acid is incorporated as a binder, ensuring tight integration of the zwitterionic cellulose nanofibers, which further improves coating uniformity, stability, and adhesion. The resulting coating exhibits superior antifogging properties, excellent adhesion resistance, and mechanical strength while remaining nontoxic to biological organisms. This coating effectively addresses practical needs in applications such as medical endoscopy, food preservation, and marine antifouling. The research offers new insights and approaches for the development of eco‐friendly and transparent protective coatings.

## Graphical abstract

A multifunctional, zwitterionic nanocellulose‐based coating is developed with lysine modification and polyacrylic acid integration, enabling robust antifogging and antifouling performance. With excellent hydrophilicity, mechanical strength, and biocompatibility, the coating shows promising potential for use in medical, food, and marine applications.

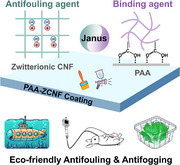

## 1 Introduction

Fogging is a common and troublesome phenomenon in daily life, generally attributed to the sudden changes in temperature and humidity that lead to the condensation of water droplets on surfaces.^[^ ^1^, ^2^, ^3^ ^]^ This phenomenon not only significantly reduces the visibility and functionality of optical devices such as camera lenses, eyeglasses, and goggles, but also poses potential safety risks due to decreased device reliability.^[^ ^4^, ^5^, ^6^, ^7^, ^8^, ^9^ ^]^ In fields like medical devices and food packaging, fogging is particularly problematic, as it can impair operational precision and visual clarity, while also providing a conducive environment for bacterial growth and biofilm formation, thereby increasing the risk of infections or accelerating food spoilage.^[^ ^10^, ^11^, ^12^, ^13^, ^14^, ^15^, ^16^, ^17^ ^]^ Consequently, the development of coatings that offer both antifogging and antifouling capabilities has become a pressing need to ensure the safety and reliability of various devices and materials.^[^ ^18^, ^19^ ^]^

Currently, antifouling and antifogging coatings are primarily categorized into hydrophilic and hydrophobic materials. Compared to hydrophobic coatings, hydrophilic coatings have attracted increasing attention due to their superior optical performance and biocompatibility.^[^ ^20^, ^21^, ^22^, ^23^, ^24^, ^25^, ^26^, ^27^, ^28^ ^]^ However, existing hydrophilic coatings still face several challenges. For instance, their antifouling performance is often inadequate and typically requires the addition of metal oxides or antifouling agents through chemical bonding. This not only increases production costs but also raises concerns regarding environmental and health impacts. Moreover, many current coating designs overlook the adhesive interactions between the coating and the substrate, which are essential for long‐term stability.^[^ ^29^, ^30^ ^]^ Particularly in humid or foggy environments, the reduced adhesion can limit the widespread application of these coatings in various practical scenarios.^[^ ^31^ ^]^ Therefore, there is an urgent need to develop a coating that offers excellent antifogging and antifouling properties to diverse materials and surfaces without significantly impacting the environment.

**Figure 1:**
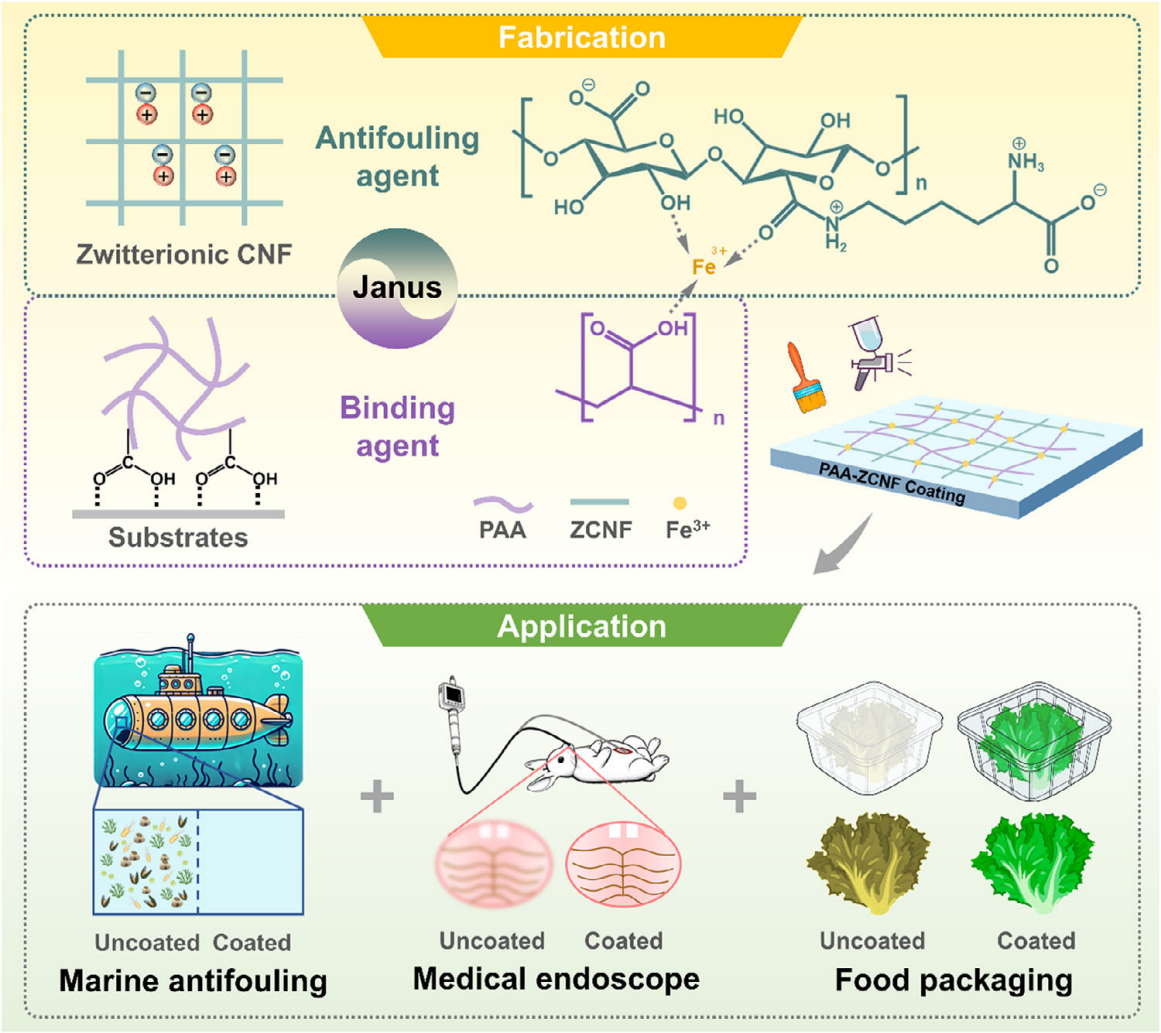
Schematic illustration of the structure and properties of the coating. A Janus coating derived from a zwitterionic nanocellulose composite material that exhibits both antifogging and antifouling properties, with the ability to be applied to a variety of surfaces, addressing the practical requirements of medical endoscopy, food packaging, and marine antifouling.

Cellulose nanofibers (CNF) are a naturally sourced nanomaterial that stands out due to their exceptional optical transparency, excellent mechanical strength, and tunable surface properties, making them promising candidates for coating applications.^[^ ^32^, ^33^, ^34^, ^35^, ^36^, ^37^, ^38^ ^]^ To address the aforementioned issues, this study presents a novel Janus coating based on CNF, featuring opposite interfacial properties at the top and bottom surfaces. Specifically, the top surface exhibits anti‐adhesive properties, while the bottom surface is engineered to enhance adhesion to the substrate. For enhanced antifouling and antifogging performance, we were inspired by the excellent antibacterial and anti‐adhesion properties of zwitterionic materials.^[^ ^39^, ^40^, ^41^, ^42^ ^]^ Lysine is used to modify CNF, introducing both positive and negative charges to create zwitterionic cellulose nanofibers (ZCNF), endowing the coating with superhydrophilicity and active antifouling capabilities without the need for harmful additives. To achieve stable adhesion on commonly used substrates (e.g., glass, polyethylene terephthalate (PET), polycarbonate), we adopt a mussel‐inspired strategy by integrating polyacrylic acid (PAA) with ZCNF through coordination cross‐linking with Fe^3+^ ions.^[^ ^43^, ^44^, ^45^, ^46^, ^47^, ^48^ ^]^ This process resulted in a coating that forms strong hydrogen bonding interactions with various substrates, significantly improving its adhesion and stability. This Janus design yields a CNF‐based coating that not only resists fogging and fouling but also maintains mechanical stability and transparency under varied environmental conditions. Furthermore, the coating's low toxicity and high biocompatibility broaden its potential application in areas such as endoscopic devices, food packaging, and marine environments (**Figure** **1**).

## 2 Results and Discussion

### 2.1 Preparation and Characterization of the ZCNF and Coating

The formation of zwitterions further enhances the hydrophilicity of the material, thereby improving the anti‐adhesion properties of the coating.^[^ ^49^ ^]^ To develop a CNF‐based antifouling coating, the synthesis of ZCNF is required first. ZCNF was produced through an EDC/NHS‐induced amidation reaction. In comparison to CNF, the infrared spectra of ZCNF displayed new peaks at 1710, 1516, and 1250 cm^−1^ (Figure S1a, Supporting Information), which are attributed to the amide I, II, and III bands, respectively.^[^ ^50^ ^]^ These bands originate from the reaction between the amino groups of lysine and the carboxyl groups on CNF. XRD analysis revealed that the crystallinity of CNF and ZCNF was very similar, at 59.5% and 58.8%, respectively, with their peak positions nearly identical. This indicates that the preparation of ZCNF does not alter the crystalline structure of CNF (Figure S1b, Supporting Information). The TEM images (Figure S1c, Supporting Information) show that ZCNF exhibits a fibrous structure similar to CNF. The mild synthesis method effectively preserved this morphology, maintaining the transparency and high strength of the nanomaterial, and ensuring its suitability for subsequent applications. Following the formation of ZCNF, the Zeta potential of CNF increased from −32.62 to −18.92 mV (measured in a pH 7.4 PBS solution with 10 mm NaCl added to standardize ionic strength). This change was attributed to the introduction of positively charged amino groups, alongside residual negatively charged carboxyl groups, leading to the formation of zwitterions. These findings collectively confirm the successful formation of ZCNF.

**Figure 2:**
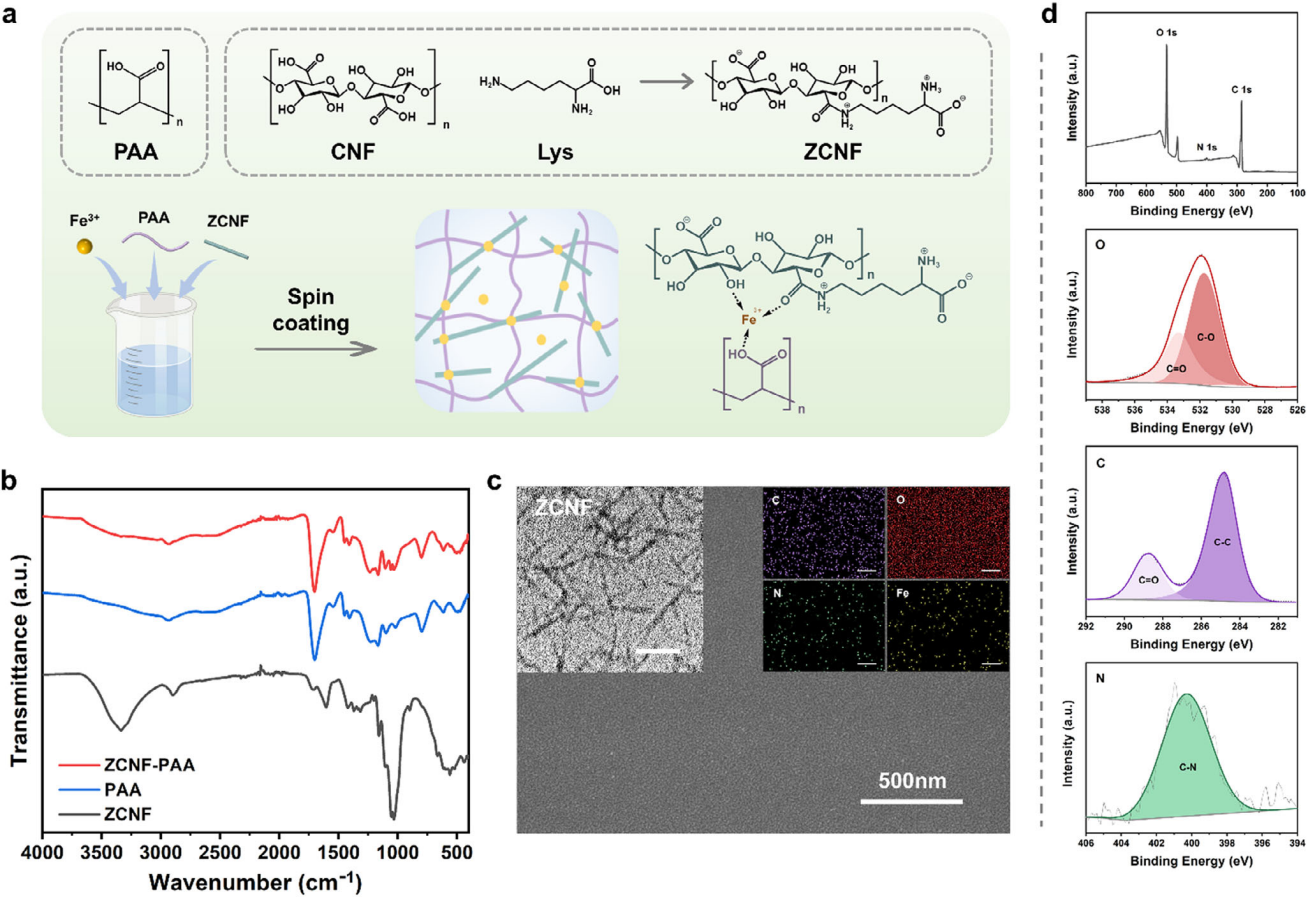
Characterization of the coating. a) Schematic illustration of the fabrication strategy. b) FTIR spectra. c) Surface morphology and distribution of elements observed by SEM. Scale bar of EDS image = 1 µm. The top left corner shows the morphology of ZCNF observed by TEM, with a scale bar of 200 nm. d) XPS survey spectra.

PAA is a commonly used hydrophilic coating material known for its excellent biocompatibility.^[^ ^51^, ^52^ ^]^ Iron ions were selected to crosslink PAA with ZCNF through coordination bonds.^[^ ^53^ ^]^ The coating synthesis process is illustrated in **Figure** **2**a. Using the orthogonal experimental method, nine coating formulations were designed (Tables S1 and S2, Supporting Information), and their water contact angle (WCA) and antifogging performances were evaluated (Table S3, Supporting Information). The results indicate that samples 2 and 8 have the lowest WCA and the best antifogging properties. Subsequently, the transmittance of the coatings was tested before and after ten abrasion cycles (Figure S2, Supporting Information). Higher transmittance indicates less wear on the coating. It was observed that coatings with a higher ZCNF content and a lower PAA content exhibited better abrasion resistance. Based on the combined results of the antifogging and abrasion resistance tests, sample 8 was selected as the final coating formulation, including concentrations of 1 mg mL^−1^ ZCNF, 25 mg mL^−1^ PAA, and 1 mg mL^−1^ Fe^3+^.

To confirm the successful synthesis of the coating, its chemical structure was first analyzed. Regarding the FTIR spectrum of ZCNF (Figure 2b), the absorption peak at 3330 cm^−1^ was attributed to the stretching vibration of hydroxyl (─OH) in ZCNF,^[^ ^54^ ^]^ and the stretching vibration peak caused by C─O─C in ZCNF appeared at 1035 cm^−1^. The broad absorption peak at 2934 cm^−1^ related to O─H stretching vibration in the carboxyl group, and the peak at 1695 cm^−1^ was assigned to the stretching vibration of C═O in the carboxyl group, both of which originate from PAA.^[^ ^55^, ^56^ ^]^ The coating contained characteristic functional groups of both ZCNF and PAA, indicating its successful synthesis. XPS analysis of the coating confirmed the presence of C, O, and N (Figure 2d). The characteristic peaks and corresponding chemical bonds are marked in the high‐resolution spectra of elements and detailed in Table S4 (Supporting Information). Consistent with the FTIR results, the XPS data also demonstrated the successful preparation of the coating.

The surface morphology was then examined, as depicted in Figure 2c. Scanning electron microscopy (SEM) revealed that the coating exhibited a smooth and flat surface, which may be attributed to the uniform dispersion of ZCNF within the coating, facilitated by its crosslinking with PAA. Additionally, cross‐sectional observations of the coatings on different substrates showed that the surfaces were also quite even, and the thickness of the coatings ranged from several tens to hundreds of micrometers (Figure S3, Supporting Information). This thickness allows the integration of nanoscale materials into the coating without increasing surface roughness. In addition, Energy Dispersive X‐ray Spectroscopy (EDS) analysis demonstrated the uniform distribution of C, O, N, and Fe elements on the coating surface (Figure 2c). These results further confirm the effective integration of ZCNF and PAA into a cohesive coating.

### 2.2 Wettability and Antifogging Performance of the Coating

**Figure 3:**
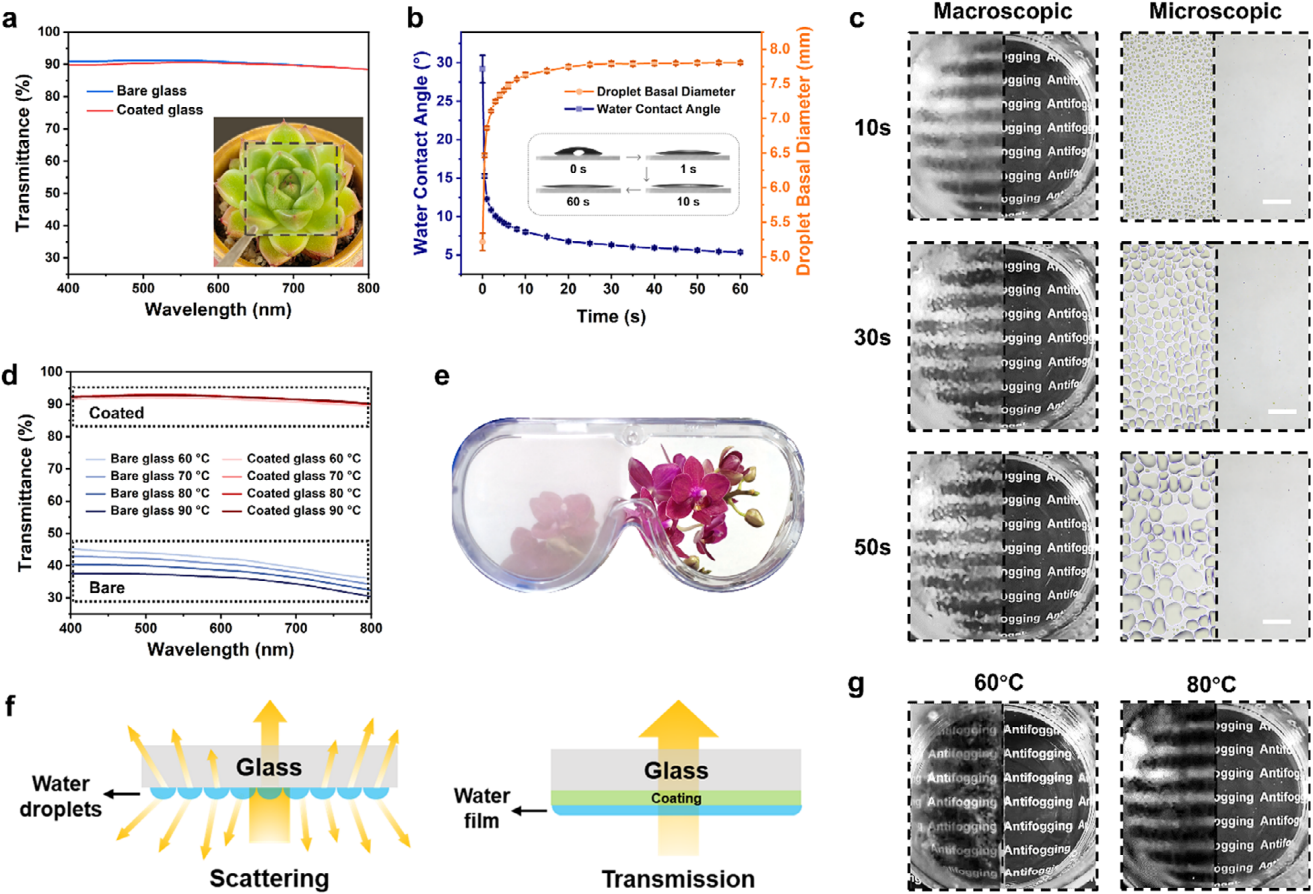
Antifogging performance of the coating. a) Transparency test results. b) Variation of WCA over time. c) Images of bare glass (left) and coated glass (right) exposure to 80 °C hot water for varying durations and corresponding microscopic images. Scale bar = 200 µm. d) Light transmittance of the coating after exposure to hot water at different temperatures. e) Antifogging effect on goggles. f) Schematic illustration of the antifogging mechanism. g) Images processed using the DCP algorithm for defogging in computer vision.

To assess the applicability of the PAA‐ZCNF coating for optical lens surfaces, we measured the light transmittance in the visible spectrum, where higher transmittance indicates greater transparency. As illustrated in **Figure** **3**a, the transmittance of the coating is nearly equivalent to that of glass. With a thickness of ≈14 µm (as shown in Figure S3, Supporting Information), the coating allows for clear visibility of the plants beneath the coating, similar to an uncoated surface. Subsequently, the wettability of the coating was evaluated by analyzing the temporal variation of the WCA. As shown in Figure 3b, upon connection with the surface, the water droplet spread rapidly, with the droplet basal diameter increasing significantly. The WCA dropped below 10° within 5 s and stabilized ≈5° after 60 s. This indicates a strong hydrophilicity of the coating. More detailed droplet morphology at various time points is illustrated in Figure S4 (Supporting Information). The water absorption capability of the coating is another crucial indicator of surface wettability. To evaluate this property, the water absorption of the coating was continuously measured over a 30 min period when exposed to hot water vapor. As illustrated in Figure S5 (Supporting Information), the water absorption approached saturation after ≈15 min, reaching 56 g m^−2^ at 30 min (with a dry coating thickness of ≈14 µm, as shown in Figure S3, Supporting Information). The high water absorption of the coating enables rapid adsorption and diffusion of water molecules on the surface, preventing localized accumulation and droplet formation, thus laying the foundation for the coating's long‐term antifogging performance.

When bare glass (Figure 3c left) and coated glass (Figure 3c right) were positioned 5 cm above hot water at 80 °C for 50 s, we recorded the condensation of vapor on the surfaces (Movie S1, Supporting Information). It was observed that the bare glass became increasingly blurred, while no fog formed on the coated surface, which remained clear throughout the observation period. Furthermore, microscopic examination revealed the formation of small droplets on the bare glass that aggregated and enlarged over time, whereas no droplets appeared on the coated glass. Figure S6 (Supporting Information) illustrates the antifogging performance at different temperatures. After steaming for 50 s at 60 °C, a faint image beneath the bare glass was visible, while at temperatures above 70 °C, recognition became increasingly difficult. In contrast, the coated glass remained clear even after heating to 90 °C. Additionally, we measured the transmittance of both coated and uncoated glass after the experiment (Figure 3d). The coated glass maintained a transmittance of ≈90%, similar to the pre‐experiment values, while the transmittance of the bare glass decreased to ≈40%, significantly impairing visibility.

Using the same methodology, the tests were conducted on PET substrates, with results presented in Figure S7 and Movie S2 (Supporting Information), confirming that the coating also exhibited high antifogging capabilities on PET. Furthermore, the coating was applied to goggles (right lens) for further evaluation. A clear contrast was observed where the uncoated side became fogged while the coated side remained clear (Figure 3e). The coating maintains strong antifogging properties across various substrates, affirming its universal applicability. The blurriness of transparent substrates can be attributed to light scattering caused by the adhered droplets. In contrast, water vapor rapidly forms a uniform film on the coated surface, allowing light to pass directly through and preserving image clarity (Figure 3f).

To further compare and validate the superiority of the proposed method, we applied the classic defogging algorithm in computer vision, Dark Channel Prior (DCP), to the left side of the comparison experiment (the region without the coating, where blur occurs). The results are shown in Figure 3g. It can be observed that when the fogging level is low (60 °C), DCP can somewhat improve the image contrast and visibility. However, when the fogging is severe (80 °C; the scene information is nearly completely lost), DCP fails to provide any effective enhancement.

To evaluate the durability of the coating, long‐term antifogging tests were conducted by continuously exposing the coated surfaces to water vapor and monitoring changes in transparency over time. As illustrated in Figure S8 (Supporting Information), water vapor condensed into droplets on bare glass or PET substrates, eventually forming larger droplets retained on the surface and impairing visibility. In contrast, the coating applied on glass maintained excellent antifogging performance for over 60 min, exhibiting minimal transparency reduction during this period and consistently providing a clear view (the bubbles observed in the images originated from heating at the bottom of the beaker and were not related to the coating performance). For the coating applied on PET, large droplets started to form after ≈40 min, likely because the coating became saturated with adsorbed water, facilitating droplet aggregation. Nevertheless, the coating on PET still demonstrated excellent antifogging properties for at least 30 min. The superior and longer‐lasting antifogging performance observed on the coated glass surfaces can be attributed to the inherently more hydrophilic nature of glass substrates, leading to improved interfacial adhesion and better wetting behavior of the coating. Consequently, water droplets could distribute more uniformly or flow more easily on the glass‐coated surfaces, allowing droplets to detach readily under gravity when reaching a certain size, thus maintaining prolonged antifogging performance.

Typically, antifogging coatings rely heavily on hydrophilic surface groups or surfactants, but these components often migrate into the bulk of the coating or undergo oxidation reactions over extended periods, causing a gradual decrease or even failure in antifogging capability. However, the introduction of ZCNF effectively addresses this issue. As presented in Figure S9 (Supporting Information), the antifogging performance of the PAA‐ZCNF coating developed in this study was compared to a commercially available antifogging coating. Results demonstrated that, after storage for one week, the developed coating still maintained antifogging capability, whereas the commercial coating lost its antifogging functionality. The sustained antifogging performance of the developed coating can be explained by two primary reasons: first, the zwitterionic functional groups are resistant to volatilization, migration, or depletion, thereby maintaining a durable hydrophilic surface for prolonged antifogging performance; second, the ZCNF exhibit excellent interfacial compatibility and strong interactions within the PAA coating matrix, providing structural stability and durability against coating failure. In summary, the introduction of ZCNF plays a crucial role in enhancing the long‐term antifogging performance of the coating.

### 2.3 Anti‐Adhesion Performance of the Coating

**Figure 4:**
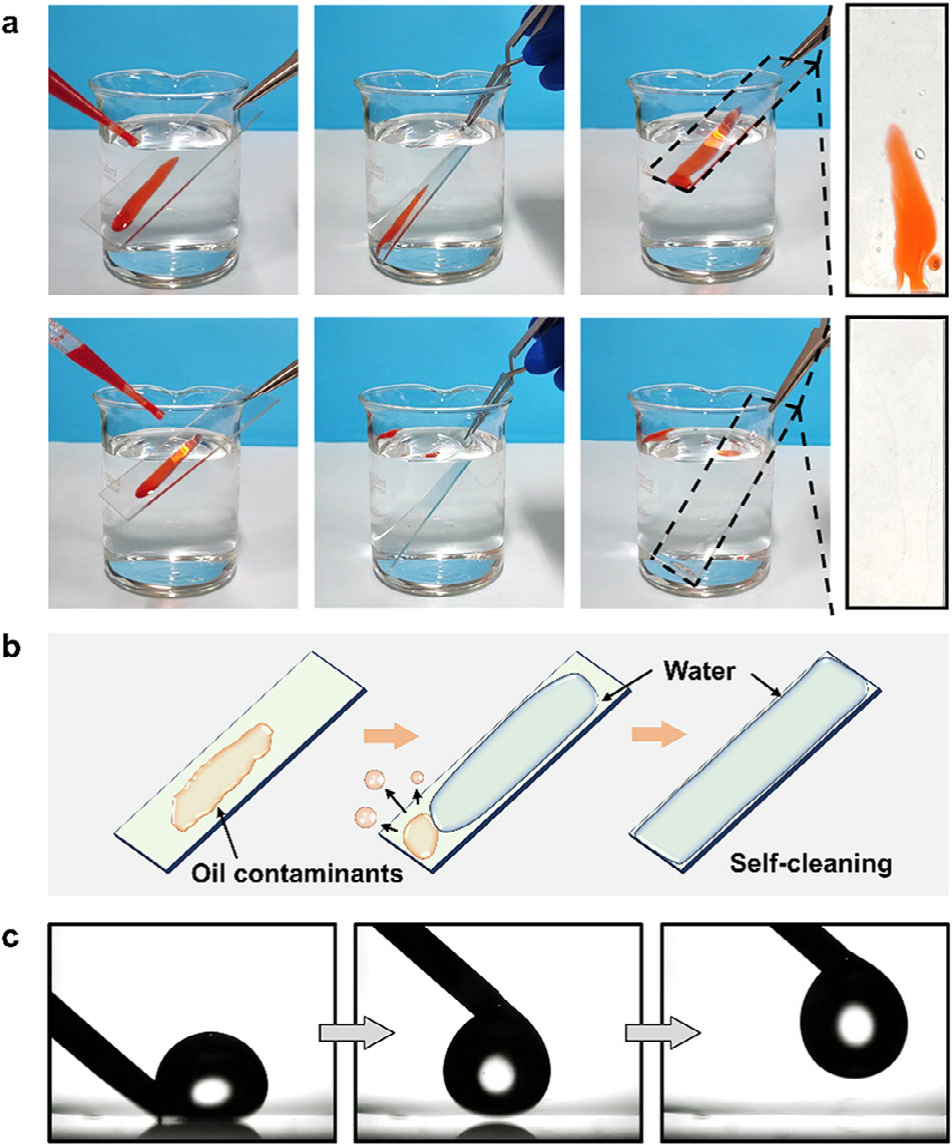
Self‐cleaning performance of the coating. a) Time‐sequenced optical photos for detachment of corn oil (dyed with Sudan III) from bare glass (top row) and coated glass (bottom row). b) Schematic illustration of the self‐cleaning mechanism. c) Surface wettability testing process: In water, a droplet of carbon tetrachloride cannot adhere to the coating, and the droplet will stay attached to the syringe needle.

The presence of contaminants on antifogging coatings can lead to erosion and adversely affect the functionality of the coated surfaces. Therefore, the development of coatings that possess both antifogging and anti‐adhesion properties is of significant importance. The resistance of the coating against oil, proteins, bacteria, and diatoms was individually evaluated. **Figure** **4** illustrates the self‐cleaning process of the coating. Upon immersion of the coating in water, the oil present on its surface detached, resulting in a clean surface without residue (Figure 4a). This phenomenon occurs because the affinity of the coating for water exceeds that for oil, leading to the rapid formation of a hydration layer on the coating, which effectively repels the oil droplets (Figure 4b).^[^ ^57^ ^]^ In contrast, oil droplets remained firmly attached to the bare glass. This observation confirms the coating's self‐cleaning capability against oil contaminants when submerged in water.

To quantitatively evaluate the coating's affinity for water, wettability tests were conducted in liquid environments. When immersed in water, the coating exhibited superoleophobicity, as organic solvents including carbon tetrachloride, bromobenzene, dichloromethane, and diiodomethane could not adhere to its surface (Figure 4c; Movies S3–S6, Supporting Information). Droplets detached from the surface upon needle retraction, even after repeated pressing against the coating. Conversely, when immersed in n‐hexane, n‐decane, n‐dodecane, xylene, or corn oil, the coating displayed superhydrophilicity (Figure S10, Supporting Information), with the WCA of ≈5°, comparable to the WCA in air. These results demonstrate the coating's strong affinity for water, which supports its underwater self‐cleaning capability.

**Figure 5:**
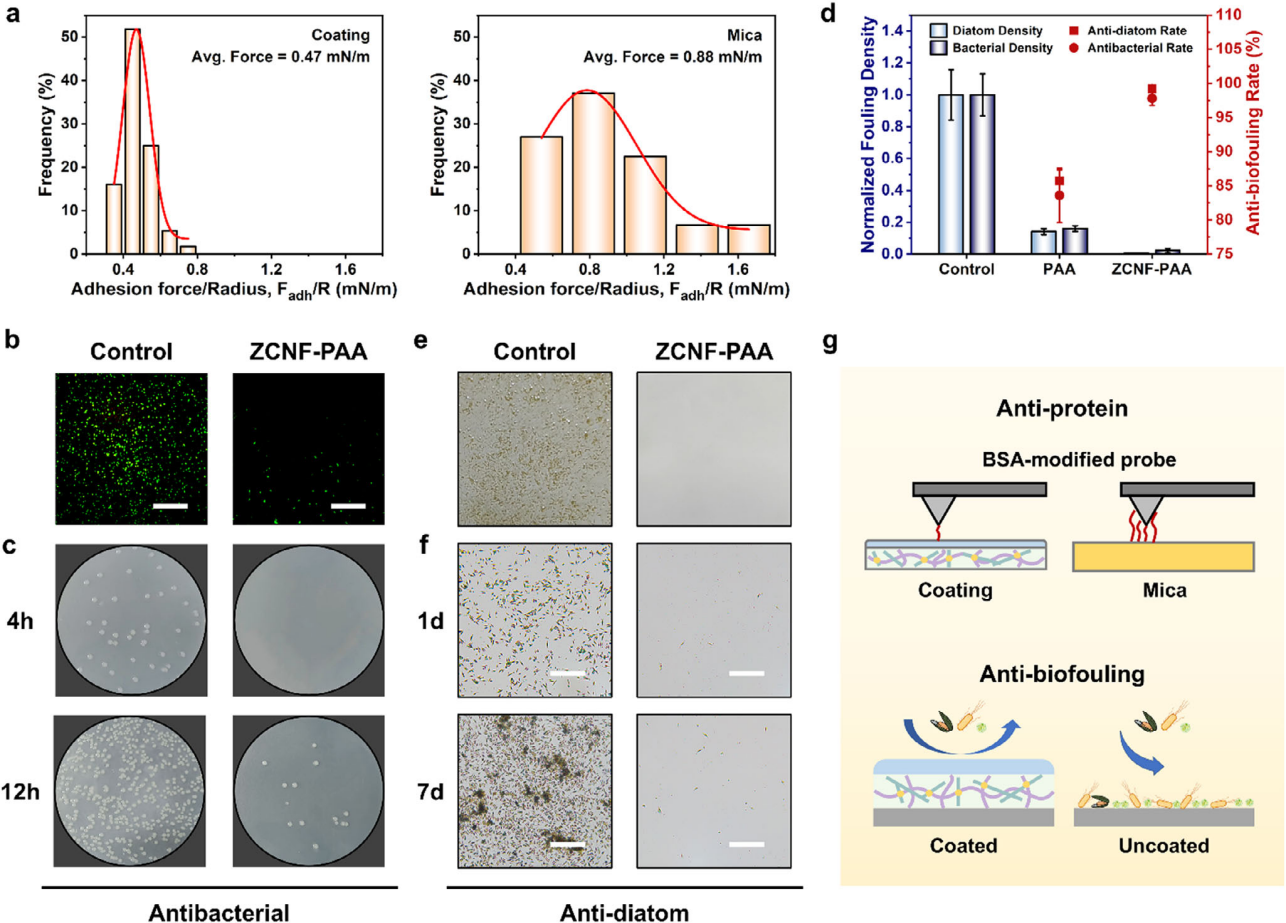
Anti‐adhesion performance of the coating. a) The adhesion force frequency between the coating and BSA measured by AFM. b) Fluorescence microscopy images of bacteria adhered on the surface after immersion for 12 h. Scale bar = 100 µm. c) Optical photos of agar plates for antibacterial tests. d) Normalized fouling density and anti‐biofouling rate. e) Optical photos of the glass and the coating after immersion in diatom suspension for 7 days. f) Microscopy images of diatom adhered to the surface. Scale bar = 200 µm. g) Schematic illustration of the anti‐adhesion mechanism.

Surface fouling typically begins with the adhesion of macromolecules, such as proteins. Therefore, measuring the interaction forces between the coating and proteins is crucial for assessing the coating's anti‐adhesive properties. Atomic force microscopy (AFM) was used to measure the interaction forces between various surfaces and bovine serum albumin (BSA) in PBS solution. Figure S11 (Supporting Information) demonstrates the successful modification of the AFM tip with BSA. As shown in **Figure** **5**a, the adhesion force of BSA to mica varied from 0.4 to 1.8 mN m^−1^, with an average value of 0.88 mN m^−1^. In contrast, the adhesive force between BSA and the coated surface was significantly reduced, ranging from 0.2 to 0.8 mN m^−1^, with an average of 0.47 mN m^−1^. The application of the coating resulted in nearly a 50% reduction in the interaction force between BSA and the mica surface, demonstrating the coating's excellent anti‐adhesive properties, which could help prevent the subsequent colonization of contaminants.

Biofouling significantly affects surfaces, as bacteria and diatoms can establish biofilms, creating stable habitats, that are difficult to remove and presenting health and safety hazards. To assess the anti‐biofouling capabilities of the coating, adhesion tests were performed with both bacteria and diatoms. *E. coli* was chosen due to its prevalent occurrence in human and environmental settings, making it a suitable model for evaluating the antibacterial efficacy of the coating. Figure 5b displays the bacterial adhesion observed under fluorescence microscopy after the coating was immersed in an *E. coli* suspension for 12 h, revealing minimal bacterial distribution, while the bare glass was extensively covered with bacteria. To quantitatively evaluate the antibacterial performance of the coating, the plate counting method was employed using photographs of *E. coli* colonies (Figure 5c). After immersing the coating in an *E. coli* suspension for 4 h, no colonies were observed on the agar plates from the coated group, while the control group exhibited the formation of several colonies. Similarly, after 12 h, the coated group showed only a few colonies, while the control group was nearly entirely covered with bacterial growth. Consequently, the antibacterial rate of the coating after 12 h was determined to be 97% (Figure 5d).

Figure 5e presents photographs taken after 7 days of immersion, clearly showing that the bare glass has turned yellow and is severely contaminated, while the coated glass remains pristine, with no visible signs of contaminants. Figure 5f presents a comparison between the coated glass and bare glass after immersion in a diatom suspension for 1 and 7 days, as observed under a microscope. After only 1 day of immersion, the bare glass exhibited a substantial attachment of diatoms, while the coated glass showed almost no diatom adhesion. With the increase in immersion duration, the growth of diatoms on the bare glass became more pronounced, with occurrences of aggregation and mortality. In contrast, the accumulation of diatoms on the coated glass was significantly lower, with a reduction in diatom density of over 99% (Figure 5d). These results indicate that the coating exhibits exceptional resistance to biological adhesion over several days, attributed to the hydrophilicity of the coating and the solvation effects of the zwitterionic groups, which facilitate the formation of a dense hydration layer on the surface.^[^ ^58^ ^]^ This layer serves as a physical barrier, preventing biofouling from contacting the surface and thereby ensuring its purity (Figure 5g).

### 2.4 Mechanical Properties of the Coating

**Figure 6:**
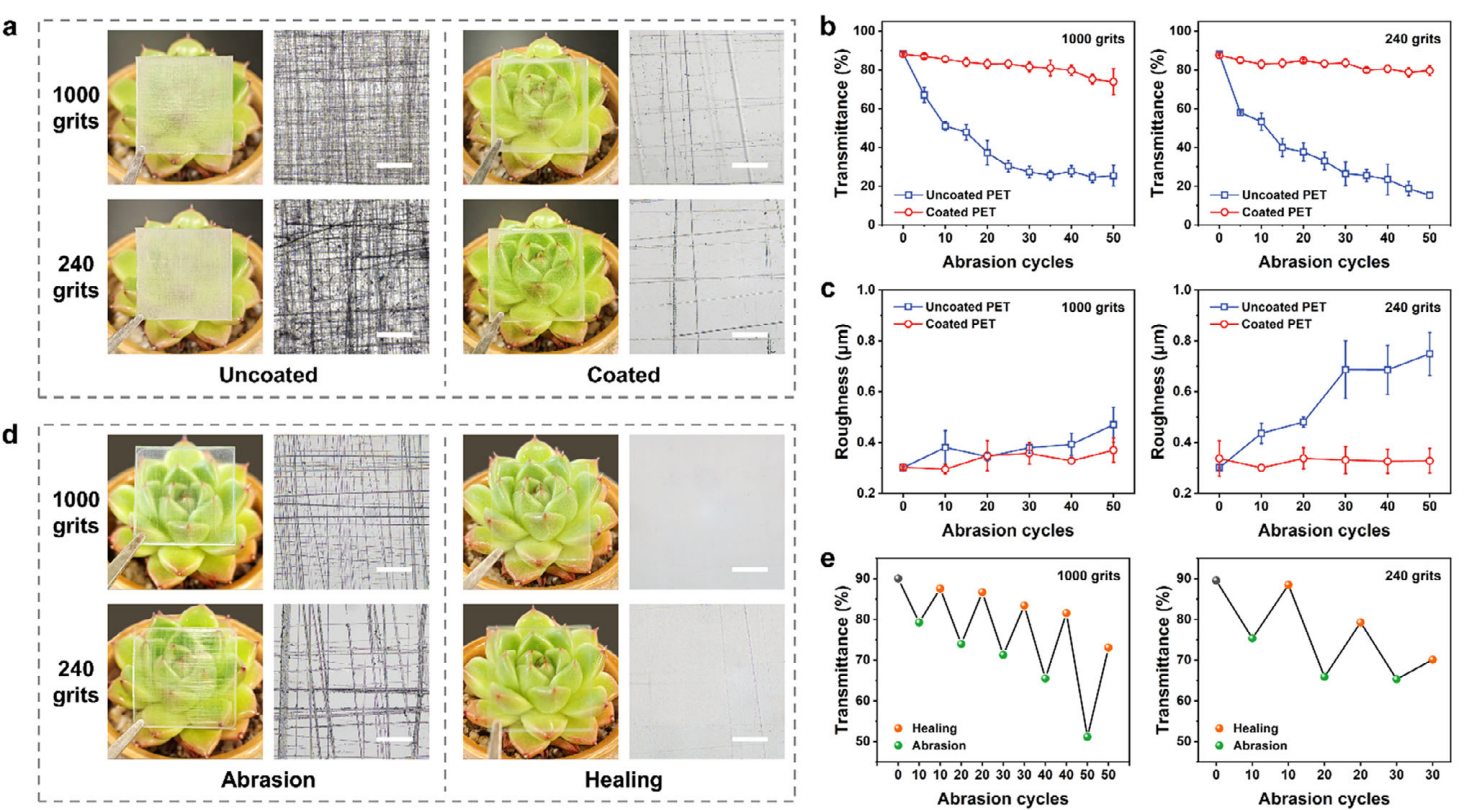
Mechanical performance of the coating. a) Observation of the underlying plants after 50 abrasion cycles on bare PET and coated PET, along with surface scratches observed under a microscope. b) Variation of light transmittance with the number of abrasion cycles. c) Change in surface roughness with the number of abrasion cycles. d) Observation of the underlying plants and surface scratches observed under a microscope after 10 abrasion cycles and subsequent healing of the coating. e) Light transmittance of the coating after multiple abrasion and healing cycles.

The coating must possess sufficient mechanical strength to ensure its durability. Therefore, its wear resistance and self‐healing properties were evaluated to ascertain its performance across various environments. To this end, a softer and more susceptible substrate, PET, was chosen to evaluate the protective function of the coating. **Figure** **6**a illustrates the observation of plants beneath bare PET and coated PET after abrasion with sandpaper. The bare PET was entirely obscured, making the plants below invisible. In contrast, the coated PET exhibited only slight wear at the edges, attributed to the edge effect of the coating, which is thinner at the perimeter. However, this minimal impact allowed for a clear view of the plants below. The wear resistance of the coating was quantitatively assessed through measurements of light transmittance (Figure 6b; Figure S12, Supporting Information). Following abrasion with fine sandpaper (1000 grit), the transmittance of bare PET plummeted from ≈90% to ≈25%. After abrasion with coarse sandpaper (240 grit), it further decreased to ≈15%, significantly impairing visibility. Conversely, the coated PET maintained a transmittance of ≈80% under both fine and coarse sandpaper abrasion, exhibiting only a slight reduction that had minimal impact on visibility. Furthermore, microscopic examination of the scratches revealed that the coated PET exhibited a substantially lower density of scratches compared to bare PET. The arithmetic average roughness (Figure 6c) and root mean square roughness (Figure S13, Supporting Information) of the coating were assessed to further investigate its wear performance. The variations in both roughness parameters exhibited similar trends. Under abrasion with fine sandpaper, the roughness of bare PET showed a slight increase, while a substantial increase was noted with coarse sandpaper, indicating that coarse sandpaper causes more severe surface wear. Whereas, the roughness of coated PET remained relatively stable before and after abrasion. Subsequently, the hardness of bare PET and coated PET was tested using the pencil hardness method, and the results are shown in Figure S14 (Supporting Information). Bare PET developed scratches even when tested with the softest 6B pencil, indicating that its hardness is lower than 6B. In contrast, the hardness of coated PET reached 4H, demonstrating a significant improvement in hardness compared to the bare substrate. This enhanced hardness is also the reason for the strong wear resistance of the coating. The high mechanical strength of nanocellulose, along with the crosslinking among the various components of the coating, contributes to its remarkable wear resistance and hardness while also providing a degree of protection to the substrate, thereby ensuring the sustained efficacy of its antifogging and antifouling properties.

Damage to the coating can lead to reduced transparency, making the exposed substrate more susceptible to contamination. Therefore, the self‐healing capability is of paramount importance. A coating with self‐healing properties can restore its original structure and performance through inherent chemical reactions or physical changes. To assess the self‐healing capability of the coating, scratches were generated using sandpaper, and after every 10 abrasion cycles, the coating was immersed in water to promote healing. To avoid any potential scratching of the substrate that might interfere with the testing of the coating, the coating was applied to hard glass. Furthermore, to facilitate the generation of scratches for self‐healing evaluation, a thin layer of the coating was applied. As illustrated in the images of the plants and microscopic views in Figure 6d, a few scratches were evident on the coating surface after ten abrasion cycles, causing some interference in the observation of the plants. Following the healing process, the coating exhibited nearly complete transparency, resembling an uncoated surface when observing the plants. Microscopic examination revealed that scratches from fine sandpaper were almost entirely eliminated, while most scratches from coarse sandpaper had diminished, with the remaining few becoming shallower, indicating partial healing. After every 10 abrasion cycles, the healing ability was assessed and quantitatively described through transmittance measurements (Figure 6e; Figure S15, Supporting Information). The healing rate of the coating was calculated by comparing the transmittance after healing with that of the initial coating, as illustrated in Figure S16 (Supporting Information). For coatings subjected to abrasion with fine sandpaper, the healing rate consistently exceeded 90% during the first four healing cycles. Additionally, the coatings that underwent abrasion with coarse sandpaper demonstrated effective healing after a single cycle. This may be due to the strong hydrogen bonding interactions and the cross‐linking effects of iron ions at the scratch interfaces, facilitating mutual attraction and adhesion.^[^ ^59^, ^60^, ^61^, ^62^ ^]^ Furthermore, a strong correlation was observed between the mechanical properties of the coating and its thickness; thicker coatings exhibited enhanced abrasion resistance and were less prone to scratching, while thinner coatings depended on their self‐healing capabilities to maintain performance. This self‐healing ability and wear resistance work synergistically, leading to a more robust coating suitable for diverse environmental applications. It should be noted that the hardness of the coating may decrease after water absorption. A pencil hardness of 2H was measured after exposure to 60 °C hot water vapor for 30 min (Figure S14, Supporting Information), indicating a slight reduction compared to the dry state. Nevertheless, the hardness remains significantly higher than that of the PET substrate and is considered sufficient for general application needs. Although reduced hardness may render the coating more susceptible to wear, its inherent self‐healing capability enables partial restoration of surface damage. This self‐repair behavior complements the coating's wear resistance, thereby enhancing its overall durability and supporting reliable performance across various demanding application scenarios.

Coating adhesion is crucial for practical application and the long‐term stability of coatings. The adhesion of the coating was evaluated on three representative substrates, including PET, epoxy resin (ER), and stainless steel (SS). As shown in Figure S17 (Supporting Information), the adhesion strength exceeded 2.4 MPa for all three substrates, with PET exhibiting the highest value of 2.91 ± 0.06 MPa. This result may be attributed to PET having a higher density of surface functional groups capable of forming strong interactions, such as hydrogen bonds, with the coating. In contrast, adhesion was relatively lower on SS surfaces. Additionally, our previous studies showed that the adhesion of nanocellulose‐based coatings was limited.^[^ ^63^ ^]^ However, the adhesion test results demonstrated that the adhesion strength of the PAA‐ZCNF coating increased by ≈1 MPa compared to the pure ZCNF coating, confirming that the adhesive effect of PAA contributes to the improved adhesion.

### 2.5 Potential Application of the Coating

**Figure 7:**
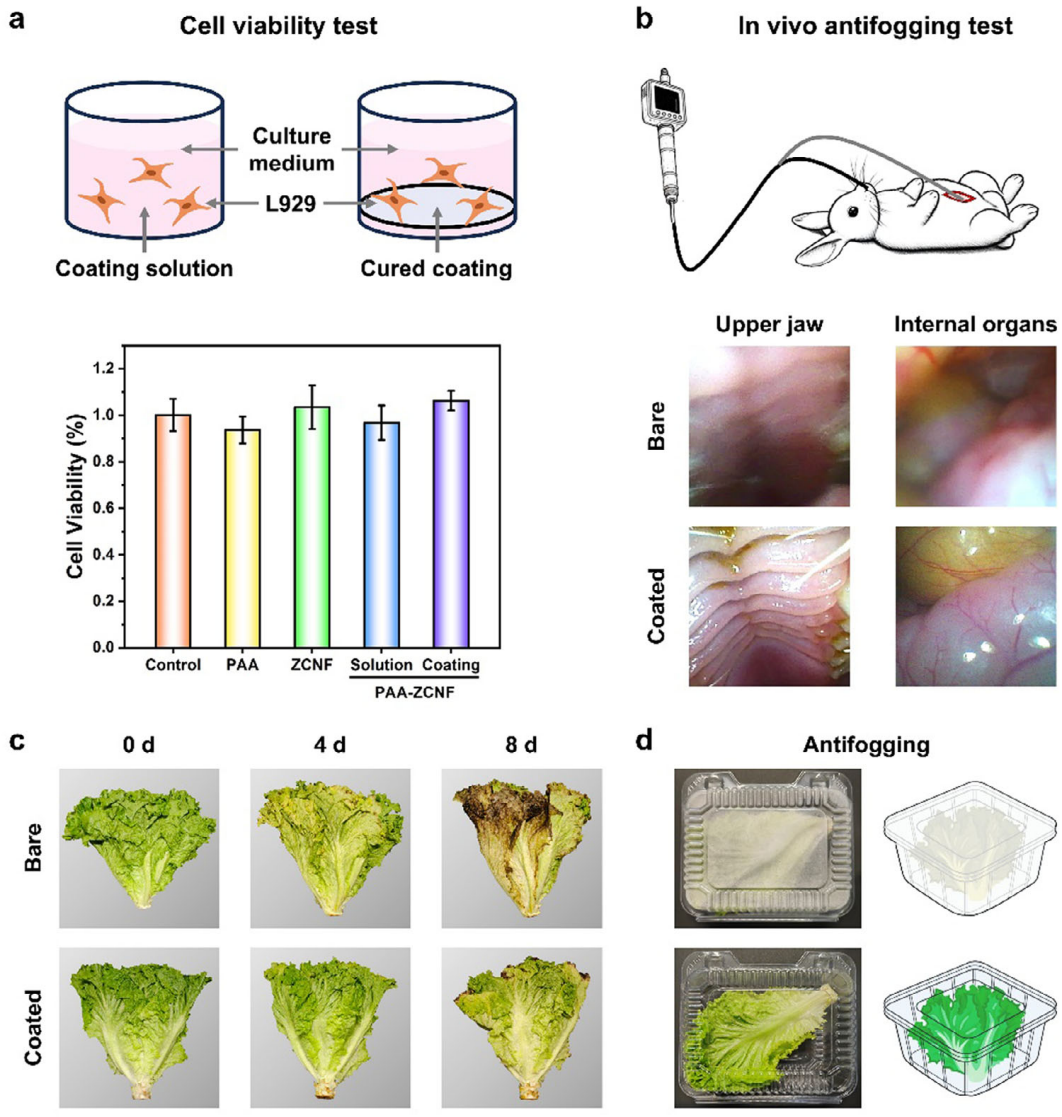
Multifunctional applications of the coating. a) L929 cell viability after co‐culturing with the coating for 24 h. b) Images of the rabbit's upper jaw and internal organs observed under an endoscope with/without the coating. Coating for food packaging: c) Lettuce preservation and d) Antifogging performance.

Building upon the demonstrated antifogging, antibiofouling, and robust mechanical properties of the coating, it is essential to evaluate its biocompatibility and potential applications. Given that the coating may come into direct contact with humans, animals, or food during use, cell viability tests were conducted on both the cured coating and its precursor solutions to assess any potential harmful effects. L929 cells, as recommended by the international standard ISO 10993–5, are widely used for evaluating the cytotoxicity of biomaterials and medical devices. Hence, they were chosen to assess the cytotoxicity of the coating in this study. As shown in **Figure** **7**a, the cell viability of all tested samples exceeded 90%, with the ZCNF and cured coatings exhibiting even higher cell viability than the control group, indicating no cytotoxicity.

Given the coating's antifogging and antiadhesion properties, it holds significant promise for applications in endoscopic devices, food packaging, and marine environments, where its protective characteristics can offer substantial benefits. Endoscopic devices are prone to fogging and protein adsorption when in contact with bodily fluids, which can obscure the view and increase the risk of surgical complications. As shown in Figure 7b and Figure S18 (Supporting Information), when an endoscope was inserted into a rabbit, the exposed lens became covered with fog, resulting in blurred images of the upper jaw and internal organs. In contrast, the coated endoscope allowed for clear visualization of these areas, maintaining a clean surface. This demonstrates the potential importance of the coating in optical and medical device applications.

The application of the coating in food packaging can help extend shelf life and prevent fogging (Figure 7c; Figure S19, Supporting Information). Lettuce stored in standard packaging bags showed noticeable yellowing, dehydration, and brown spots on the 4th day, with significant decay and browning by the 8th day. In contrast, lettuce stored in coated packaging bags exhibited only slight yellowing and dehydration at the leaf edges on day 4, and after 8 days, only minor brown spots appeared at the edges, similar to the control group on day 4. Furthermore, to quantitatively analyze the color changes in the lettuce, hue and saturation were measured from the images. The experimental group showed a smaller variation compared to the control group (Figure S20a, Supporting Information). Analysis of the lettuce's weight loss (Figure S20b, Supporting Information) revealed that the control group lost 15% of its mass, while the experimental group lost only 9%, indicating that the coating slows down the rotting process and reduces water loss. Due to the evaporation of moisture, food packaging is prone to fogging, which can hinder observation. As shown in Figure 7d lettuce stored in a standard container was covered in water droplets under low temperature, whereas the coating effectively prevented this phenomenon.

Finally, the coating's capability to mitigate fouling in a marine environment was investigated. Commercially available epoxy panels modified with or without the antifouling coating were immersed for 40 days at 0.5 m below sea level. Evaluation of fouling was performed at different time points via visual inspection of the panels. As seen in Figure S21 (Supporting Information), the coated samples remained nearly clean during the first 20 days of marine exposure, effectively inhibiting the attachment of marine organisms. Although partial biofouling was observed on day 40, the fouling degree on the coated surfaces was still significantly lower than that of the uncoated control group. As shown in Figure S22 (Supporting Information), statistical analysis of fouling coverage revealed that after 20 days of immersion, the fouling coverage of the coated samples was reduced by ≈65% compared to the uncoated samples; after 40 days, the coating still retained a fouling suppression efficiency of ≈51%. These findings clearly demonstrate the coating's good stability and antifouling capability in highly fouling marine environments, highlighting its potential for application in marine equipment.

## 3 Conclusion

This study presents a novel Janus coating based on CNF, which incorporates both antifouling agents and a binding component. The enhanced hydrophilicity and zwitterionic characteristics of ZCNF impart excellent antifouling and antifogging properties to the coating, along with high mechanical strength. PAA acts as a critical binder, improving the coating's uniformity and stability, while facilitating strong hydrogen bonding with substrates for secure adhesion in humid or underwater environments. This also enables easy application via methods such as spraying or brushing. The unique combination of these features contributes to the coating's outstanding performance. Protein adhesion tests demonstrate significantly reduced interactions between the coating and contaminants, highlighting its effectiveness in preventing oil adhesion and enhancing self‐cleaning properties. Notably, the coating exhibits over 97% resistance to biofouling even after prolonged exposure. Furthermore, it shows outstanding mechanical durability, including wear resistance and self‐healing capabilities, ensuring sustained antifogging and antifouling performance under harsh conditions. The coating's eco‐friendly nature, biocompatibility, and non‐cytotoxicity highlight its potential for sustainable applications in medical devices, food packaging, and marine environments. Overall, this study presents a versatile and high‐performance coating strategy that offers a reliable solution to address fogging and fouling challenges in practical applications.

## 4 Experimental Section

### Materials

PAA, FeCl_3_·6H_2_O, 2,2,6,6‐tetramethylpiperidine‐1‐oxyl radical (TEMPO), NaBr, NaOH, NaClO, 2‐Morpholinoethanesulfonic acid (MES), 1‐(3‐Dimethylaminopropyl)‐3‐ethylcarbodiimide hydrochloride (EDC), N‐Hydroxy succinimide (NHS), lysine, BSA, ethanol, propylene glycol, polysorbate and Sudan III were purchased from Shanghai Aladdin Biochemical Technology Co., Ltd. (China). Sodium borohydride (NaBH_4_) was purchased from Sinopharm. The Superbrilliant CCK8 Cell Proliferation and Toxicity Detection Kit was purchased from Zhongshi Gene Technology (Tianjin) Co., Ltd. *Escherichia coli* (*E. coli*) was purchased from China General Microbiological Culture Collection Center. *Nitzschia closterium f. minutissima* (*N. closterium f. minutissima*) and Guillard's F/2 culture medium were purchased from Shanghai Guangyu Biological Technology Co., Ltd. (China).

### Preparation of ZCNF

The TEMPO‐mediated oxidation of pulp was carried out at a 2 wt.% concentration, following established procedures.^[^ ^64^ ^]^ Briefly, 0.1 mmol TEMPO and 1 mmol NaBr per gram of pulp were dissolved in deionized water, and the pH was adjusted to 10 using NaOH. Pulp was added to the solution and stirred for 1 h to allow chemical impregnation via fiber swelling. The oxidation reaction was initiated by adding 7.5 mmol NaClO per gram of pulp at room temperature, maintaining the pH at 10 until NaOH consumption ceased. The mixture was vacuum‐filtered to separate the oxidized pulp, which was then washed with pure water. CNF were produced by mechanically disintegrating the oxidized pulp using a high‐pressure homogenizer (AH‐NANO, ATS Engineering Limited, China). The product was diluted to 0.2 wt.% and mixed with MES, EDC, and NHS in a mass ratio of 1:1.5:1:1 with respect to CNF. The mixture was stirred for 2 h to activate the carboxyl groups fully. Solutions of lysine dissolved in PBS at the same mass as the CNF, were then added, with stirring continuing overnight. The resulting mixtures were dialyzed for two days to obtain lysine‐modified zwitterionic CNF. The chemical constitution of ZCNF was characterized by Fourier transform infrared spectroscopy (FTIR, Nicolet IS50, Thermo Fisher), while its microstructure was examined using transmission electron microscopy (TEM, JEM 1400 Flash, JEOL). The crystallization was determined by X‐ray diffractometer (XRD) (D8‐Focus, Broker AXS) using CuKα radiation in the 2θ range from 5° to 40°, and the crystallinity index (CI) was calculated by Segal equation: *CI* = (*I*_200_ − *I*_am_) / *I*_200_ × 100%, where *I*_200_ is the maximum intensity of (200) crystal plane at 22.4° and *I*_am_ is the intensity of amorphous diffraction, which is taken ≈18.5°. A Zetasizer (Nano ZS, Malvern) was used to determine the zeta potential.

### Coating Preparation and Characterization

The optimal coating formulation was determined using the orthogonal experimental design, with the composition and content of the coatings provided in Tables S1 and S2 (Supporting Information). Glass and PET were used as substrates, which were ultrasonically cleaned with ethanol and deionized water. The coating solution was then spin‐coated onto the substrates at 1500 rpm for 20 s to produce the composite coating PAA‐ZCNF. To facilitate the application of the coating on different materials with various shapes, the solvent of the coating solution was optimized without altering the concentration or proportion of the coating components (shown in Table S5, Supporting Information). This optimization allows the coating to be applied by spraying or brushing onto various surfaces and enables its use in subsequent animal and food packaging experiments. The chemical construction of the coating was analyzed using FTIR and X‐ray photoelectron spectroscopy (XPS, K‐Alpha+, Thermo Fisher), while its microstructure was observed with SEM (Regulus 8100, Hitachi). The transmittance of the coating was measured by a UV–vis spectrophotometer (UV‐2700i, Shimadzu). The wettability was determined by WCA measurements (Theta Lite, Biolin), and the time‐dependent contact angles were collected over 60 s. The surface roughness was evaluated using a surface roughness tester (SJ‐210, Mitutoyo). The water absorption test method for the coating was based on previous literature.^[^ ^5^ ^]^ Specifically, the water absorption of the coating was measured by applying the coating to the glass and continuously exposing it to 60 °C water vapor. The increase in the coating's weight, representing the amount of water absorbed, was measured at regular intervals. The adhesion strength of the coating was measured by an automatic tester (PosiTest, Model AT‐A) according to ASTM D4541‐09. The hardness of the coating was evaluated with a HST‐5608 electric pencil hardness tester (Shenzhen Xiangbang Automation Instrument Equipment Co., Ltd.) according to ASTM D3363. The applied pressure during the test was 500 g.

### Antifogging Measurement

The antifogging property was tested under hot‐steam conditions.^[^ ^65^ ^]^ Bare glass and coated glass were placed 5 cm above hot water (60–90 °C) for 40 s to allow steam condensation on the surface. The antifogging performance was then evaluated by observing the transparency and monitoring the formation of water droplets under a microscope (CX‐23, Olympus) to understand the antifogging mechanism. The same method was used to test the antifogging capability of the coating on a PET substrate to verify its applicability across different substrates. The coating applied on glass and PET substrates was continuously exposed to water vapor at 60 °C for 60 min. Photos were taken every 10 min to record the coating conditions, and the optical transmittance was measured at each interval. The commercial antifogging coating (Zhejiang Yuewei Advanced Materials Co., Ltd.) and the PAA‐ZCNF coating were separately applied to glass slides. After being stored at room temperature for different periods (0, 3, and 7 days), the antifogging performance of the coatings was tested. Photographs were taken to record the fogging condition of the coatings after exposure to hot steam at 80 °C for 50 s.

### Self‐Cleaning Test

Sudan III‐stained soybean oil was first dropped onto the coating surface. The coating was then immersed in water for a few seconds before being removed, and the residual oil on the surface was observed to assess the self‐cleaning ability of the coating. To quantitatively assess the anti‐adhesion performance of the coating toward oil phases, the contact angles of the coating in underwater oil (carbon tetrachloride, bromobenzene, dichloromethane, and diiodomethane) were measured. Additionally, the WCA of the coating in various oil phases (n‐hexane, n‐decane, n‐dodecane, xylene, and corn oil) were also evaluated.

### Anti‐Protein Test

The adhesion force between the coating and the contaminant (BSA) was quantitatively measured using an AFM. To functionalize BSA on AFM probe, the AFM probe (B triangular NPG‐10, Bruker) was treated in UV‐Ozone (UVO machine, CIF) for 20 min, followed by an immersion in a mixed solution of BSA (8 mg mL^−1^) and NaBH_4_ (100 mm) at a volume ratio of 1:1 for 12 h.^[^ ^66^ ^]^ Then the AFM probe was washed with water and ethanol to remove unreacted chemicals. An MFP‐3D AFM (Asylum Research, Santa Barbara, CA, USA) was used to measure the interaction forces between the BSA functionalized AFM probe and the coating in PBS buffer (10 mm).^[^ ^67^ ^]^ The cantilever's spring constant was determined using the Hutter and Bechhoefer method.^[^ ^68^ ^]^ In a typical force measurement, the AFM probe was driven to approach the substrate at a constant velocity until reaching a desired force (10 nN), after which the probe was retracted from the substrate. The velocity was set as 1 µm s^−1^ in the force measurements to minimize hydrodynamic effect. 100 approach‐retraction force cycles in total were performed in a 2 × 2 µm^2^ area and at least three different areas were measured for each substrate.

### Antibacterial Test

The coatings were immersed in 15 mL of an *E. coli* suspension expressing green fluorescent protein (≈10^6^ CFU) and incubated at 37 °C for 12 h. After incubation, the coatings were rinsed three times with PBS buffer to remove loosely adhered bacteria, and the rinsing solution was discarded. Some of the coatings were then observed under a fluorescence microscope (BX‐43, Olympus), and the bacterial density on the surface was quantified. The remaining coatings were repeatedly rinsed to completely detach the adhered bacteria. Then, 100 µL of the diluted bacterial suspension from the rinsing solution was spread onto Luria‐Bertani agar plates and incubated for another 18 h at 37 °C, followed by counting the number of colonies on the plates. The antibacterial experiments were performed in triplicate.

### Anti‐Diatom Test

Guillard's F/2 culture medium was used to cultivate *N. closterium f. minutissima* (a common benthic marine biofouling microalga) in an illuminated incubator set to 20 °C with a 12‐h light‐dark cycle. Coated glass slides were immersed in 30 mL of a diatom suspension (≈10^8^ cells per mL). After one week of cultivation, the slides were removed, and any loosely attached diatoms were rinsed off with seawater. The diatoms that remained on the coatings were then examined under an optical microscope, and their density was measured in five randomly selected areas.

The anti‐biofouling rate (*R_a_*) is calculated by the following formula:

(1) $$R_{a}=\frac{D_{b}-D_{c}}{D_{b}}\times 100\%$$

*D*_b_ and *D*_c_ respectively refer to the biofouling density corresponding to the bared control group and the coated experimental group.

### Mechanical Properties Measurement

A wear resistance test and self‐healing experiment were carried out on the coated PET. The coatings were abraded using fine sandpaper (1000 grit) and coarse sandpaper (240 grit). The coating surface was placed face down on the sandpaper and pressed with a 50 g weight. The coating was moved 10 cm in a straight line, then rotated 90°, and moved another 10 cm. This procedure constituted one abrasion cycle. For the abrasion resistance test, each coating underwent 50 abrasion cycles, with transmittance measured every 5 cycles and roughness tested every 10 cycles. In the self‐healing experiment, a healing process was performed every 10 abrasion cycles by immersing the coating in water for 1 s. Transmittance and roughness were recorded before and after each healing process. Each experiment was repeated with three samples.

### Biosafety Assessment

The Cell Counting Kit‐8 (CCK‐8) assay was used to evaluate the cytotoxicity of the coating and its components. As recommended by ISO 10993–5:2009, L929 cells were seeded at a density of 1 × 10^4^ cells per mL in complete DMEM medium (DMEM + 10% FBS + 1% P/S) and incubated overnight at 37 °C with 5% CO_2_. Different samples (10 vol% of the culture medium) and coated glass slides were then added, and the cultures were incubated for an additional 24 h. After incubation, CCK‐8 working solution (prepared at a 1:10 ratio of CCK‐8 solution to medium) was added, and the cells were further incubated for 3 h. Absorbance at 450 nm was measured to assess cell viability, which was calculated using the following formula:

(2) $$Cell\;viability=\frac{OD_{2}-OD_{0}}{OD_{1}-OD_{0}}\times 100\%$$

where *OD*_0_, *OD*_1_, and *OD*_2_ represent the blank group (medium only), the control group (cells without sample), and the experimental group, respectively.

### In Vivo Application

All procedures involving animals were conducted in accordance with the Tianjin Experimental Animal Management Ordinance, China. A rabbit (2.2 kg) was selected to assess the potential in vivo application of the coating. An anesthetic was administered via intravenous injection into the rabbit's ear, and once the animal was immobilized, a digital endoscope (MJ‐KR‐4.8D, Jiangsu Maijun Medical Technology Co., Ltd), either coated or uncoated, was used to observe and photograph the oral cavity, esophagus, and abdominal cavity.

### Food Packaging Application

The coating was sprayed onto food packaging (PET boxes and bags), and the fresh lettuce was placed inside the packaging, with three replicates. For the antifogging test, the boxes containing lettuce were stored in a refrigerator at 4 °C for 1 h, then removed for observation and photography. For the preservation test, the lettuce was placed in the bags and stored at room temperature for a certain period. The lettuce was photographed and weighed on days 0, 4, and 8 to assess the extent of spoilage. Hue and saturation changes in the lettuce photographs were analyzed using MATLAB software.

### Marine Field Test

The marine field tests were conducted over a 40‐day period from June to July in the Bohai Sea (117°46′E, 39°43′N), Tianjin, China. Coatings were applied to epoxy resin panels (50 mm × 50 mm × 3 mm), which were then immersed at a depth of 0.5 m below sea level, with two replicates. At specified intervals, the panels were retrieved, rinsed with seawater to remove loosely attached fouling, and photographed. The fouling area was quantified using ImageJ software. To account for edge effects, only the central 40 mm × 40 mm area of each panel was analyzed.

## Conflict of Interest

The authors declare no conflict of interest.

## Supporting information



Supporting Information

Supplemental Movie 1

Supplemental Movie 2

Supplemental Movie 3

Supplemental Movie 4

Supplemental Movie 5

Supplemental Movie 6

## Data Availability

The data that support the findings of this study are available from the corresponding author upon reasonable request.
