# Supplementary material for: Robust Antifogging and Antifouling Coating Tailored with Zwitterionic Nanocellulose for Multi‐Functional Applications
Source: Adv Sci (Weinh). 2025 Sep 10;12(39):e09204. doi: 10.1002/advs.202509204 (PMC12533154; doi:10.1002/advs.202509204)
Supplement: Supplementary file 1 — Supporting Information [file ADVS-12-e09204-s007.docx]

**Supporting Information of**

**Robust Antifogging and Antifouling Coating Tailored with** **Zwitterionic Nanocellulose for Multi-Functional Applications**

*Yanyi Duan ^a^, Jiangjiexing Wu ^b*^, Aihua Qiao ^b^, Zheng Zhang ^c^, Jiaxin Shang ^c^, Xiaohui Mao ^d^, Xiaohui Yan ^e^, Nan Wang ^e^, Peng Zhong ^e^, Xiaobo Li ^b^, Xavier Banquy ^f*^, Wei Qi ^a^, Rongxin Su ^a,b*^*

^a^ State Key Laboratory of Chemical Engineering, Tianjin Key Laboratory of Membrane Science and Desalination Technology, School of Chemical Engineering and Technology, Tianjin University, Tianjin 300072, PR China

^b^ School of Marine Science and Technology, Tianjin University, Tianjin 300072, PR China

^c^ Tianjin Stomatological Hospital, School of Medicine, Nankai University, Tianjin, China

^d^ College of Materials Science and Engineering, Donghua University, 2999 North Renmin Road, Shanghai, 201620, P. R. China

^e^ State Key Laboratory of Chinese Medicine Modernization, Tianjin University of Traditional Chinese Medicine, 10 Poyanghu Road, Tianjin 301617, PR China

^f^ Faculty of Pharmacy, Université de Montréal, Montréal, H3T 1J4, QC, Canada

* Corresponding authors:

Jiangjiexing Wu, E-mail: wjjx1987@tju.edu.cn

Xavier Banquy, E-mail: xavier.banquy@umontreal.ca

Rongxin Su, E-mail: surx@tju.edu.cn

**Table of Contents**

**Table S1.** Level factor table of various parameters.

**Table S2.** Orthogonal experiment table of 9 as-prepared samples.

**Table S3.** Water contact angle and antifogging performance of the samples.

**Table S4.** Concentrations of coating components and solvent ratios in the optimized coating.

**Table S5.** Peak area ratio of each chemical bond.

**Table S6.** The antifouling performance of hydrophilic coatings reported in recent five years.

**Figure S1.** Characterization of zwitterionic cellulose nanofibers.

**Figure S2.** Light transmittance of 9 as-prepared samples before (left) and after (right) abrasion.

**Figure S3.** Thickness of the coating on different substrates.

**Figure S4.** Variation of contact angle over time.

**Figure S5.** Water absorption of the coating over 30 min.

**Figure S6.** Antifogging performance (exposure to hot water 30 s) of the coating at different temperatures.

**Figure S7.** Antifogging performance (exposure to 80 °C hot water) of the coating on PET substrate.

**Figure S8.** Long-lasting antifogging performance of the coating on a) glass substrate and b) PET substrate.

**Figure S9.** Antifogging performance of the PAA-ZCNF coating and commercial coating after storage for 0, 3, and 7 days.

**Figure S10.** Water contact angle of the coating in different solvents.

**Figure S11.** The elemental distribution map of the BSA-modified AFM probe.

**Figure S12.** Comparison of light transmittance between coated and uncoated PET after abrasion.

**Figure S13.** Comparison of root mean square roughness between coated and uncoated PET after abrasion.

**Figure S14.** Pencil hardness of PET and coated PET.

**Figure S15.** Light transmittance of the coating after multiple abrasion and healing cycles.

**Figure S16.** Healing rate of the coating.

**Figure S17.** Adhesion force of the pure ZCNF coating and PAA-ZCNF coating on various substrates.

**Figure S18.** Photographs of the animal experimental procedure.

**Figure S19.** The lettuce in the packaging.

**Figure S20.** Changes of lettuce with preservation time.

**Figure S21.** Marine field test.

**Figure S22.** Statistical analysis of the fouling coverage area on panels at different time points in the marine field test.

**Table S1.** Level factor table of various parameters.

| **Level** | **Factor** | | |
| --- | --- | --- | --- |
|  | **ZCNF concentration**  **(A) (%)** | **Fe^3+^ concentration**  **(B) (mg/ml)** | **PAA concentration**  **(C) (mg/ml)** |
| 1 | 0.01 (A1) | 0.1 (B1) | 25 (C1) |
| 2 | 0.03 (A2) | 1 (B2) | 75 (C2) |
| 3 | 0.1 (A3) | 10 (B3) | 150 (C3) |

**Table S2.** Orthogonal experiment table of 9 as-prepared samples.

| **Serial**  **number** | **ZCNF concentration**  **(A) (%)** | **Fe^3+^ concentration**  **(B) (mg/mL)** | **PAA concentration**  **(C) (mg/mL)** |
| --- | --- | --- | --- |
| 1 | 0.01 | 0.1 | 25 |
| 2 | 0.01 | 1 | 75 |
| 3 | 0.01 | 10 | 150 |
| 4 | 0.03 | 0.1 | 75 |
| 5 | 0.03 | 1 | 150 |
| 6 | 0.03 | 10 | 25 |
| 7 | 0.1 | 0.1 | 150 |
| 8 | 0.1 | 1 | 25 |
| 9 | 0.1 | 10 | 75 |

**Table S3.** Water contact angle and antifogging performance of the samples.

| **Serial number** | 1 | 2 | 3 | 4 | 5 | 6 | 7 | 8 | 9 |
| --- | --- | --- | --- | --- | --- | --- | --- | --- | --- |
| **WCA (°)** | 11.06 | 8.84 | 22.19 | 35.93 | 13.2 | 30.15 | 24.57 | 9.15 | 33.7 |
| **Antifogging**  **Performance** | **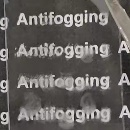** | **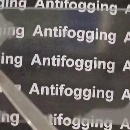** | **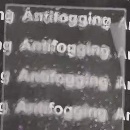** | **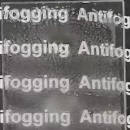** | **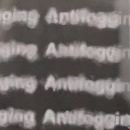** | **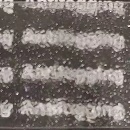** | **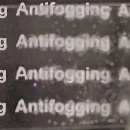** | **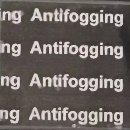** | **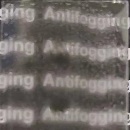** |

**Table S4.** Peak area ratio of each chemical bond.

| **Elements** | **Atomic %** | **Bonds** | **Peak Area Ratio** |
| --- | --- | --- | --- |
| C 1s | 63.56 % | C―C | 0.75 |
|  |  | C＝O | 0.25 |
| O 1s | 34.76 % | C―O | 0.66 |
|  |  | C＝O | 0.34 |
| N 1s | 1.67 % | C―N | 1 |

**Table S5.** Concentrations of coating components and solvent ratios in the optimized coating.

| **Components (mg/mL)** | | | | **Solvent (%)** | | |
| --- | --- | --- | --- | --- | --- | --- |
| ZCNF | PAA | Fe^3+^ | Polysorbate | Water | Ethanol | Propylene  glycol |
| 1 | 25 | 1 | 1 | 25 | 70 | 5 |

**Table S6.** The antifouling performance of hydrophilic coatings reported in recent five years.

| **The composition of coatings** | **Duration of anti-diatom test (days)** | **Anti-diatom rate** | **Duration of anti-bacteria test (hours)** | **Anti-bacteria rate** | **Duration of marine field test (days)** | **Marine field antifouling performance** |
| --- | --- | --- | --- | --- | --- | --- |
| This work | 1 | 99.9% | 4 | 100% | 40 | 51% |
|  | 7 | 99.2% | 12 | 97.8% |  |  |
| Poly (carboxybetaine acrylamide) hydrogel coating^[1]^ | 14 | 81% | / | / | 45 | 90 points (ASTM D3623-78a scoring system) |
| Coatings based on amide derivatives with metal oxide antifouling agents ^[2]^ | 7 | 80% | 24 | 99% | 240 | Good antifouling activity |
| Dicyclopentenyl/carboxybetaine-containing copolymers^[3]^ | 90 min | 99% | / | / | 5 | 77% |
| Zwitterionic hydrogel coating modified by metal-organic framework^[4]^ | 1 | 99% | 4 | 100% | / | / |
| Polyampholyte hydrophilic film^[5]^ | / | / | 24 | 100% | / | / |
| Polyphosphoester polymer brushes^[6]^ | / | / | 2 | 98.8% | / | / |
| Zwitterionic Polymeric Sulfur Ylides^[7]^ | / | / | 4 | 80% | / | / |
| Multilayer bioadhesive patch containing a zwitterionic-interpenetrated elastomer layer^[8]^ | / | / | 24 | 99.9% | / | / |
| PAA/PSBMA/Al(OH)_3_ composite hydrogel coating^[9]^ | / | / | 6 | 100% | / | / |
| Polyelectrolyte complex films^[10]^ | 1 | 100% | / | / | / | / |
| Poly(vinyl alcohol)/glycerol–tannic acid/Cu^2+^ hydrogel^[11]^ | 5 | Low density |  |  | / | / |


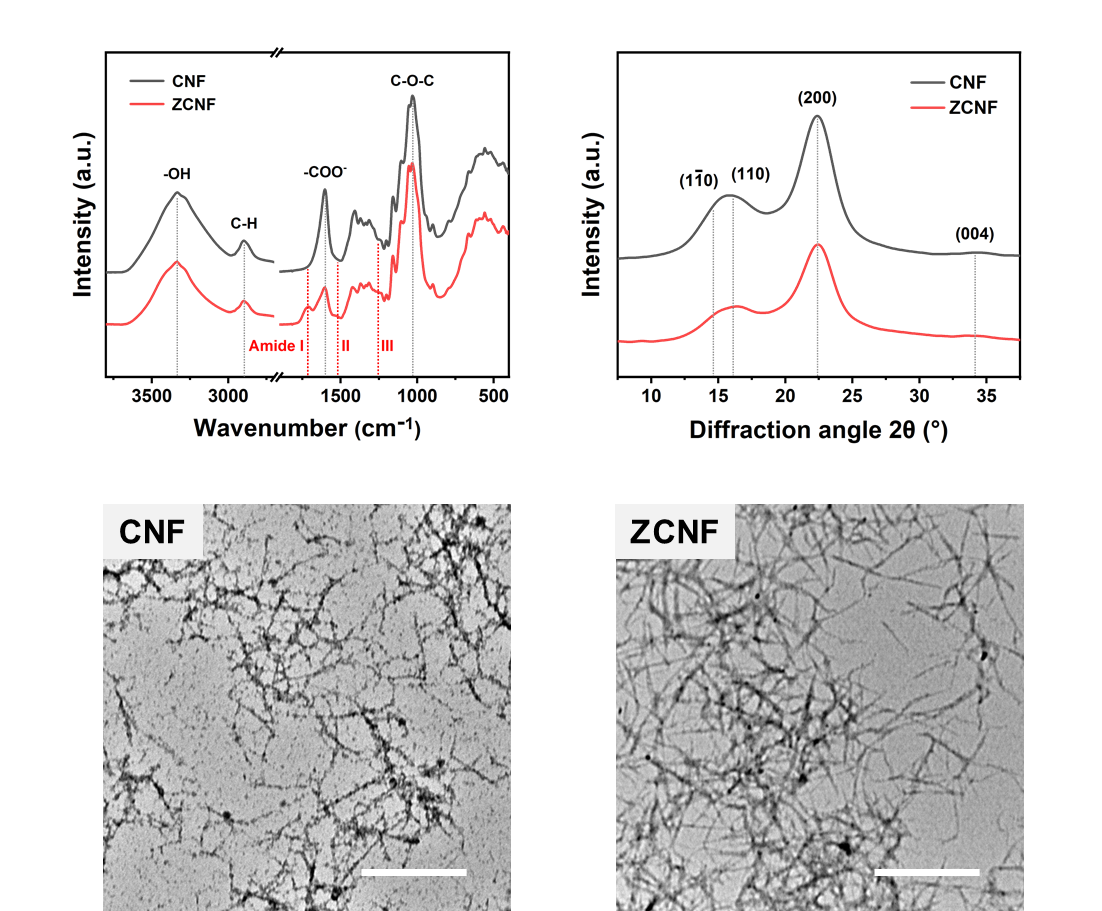


**Figure S1.** Characterization of zwitterionic cellulose nanofibers. Scale bar = 500 nm.


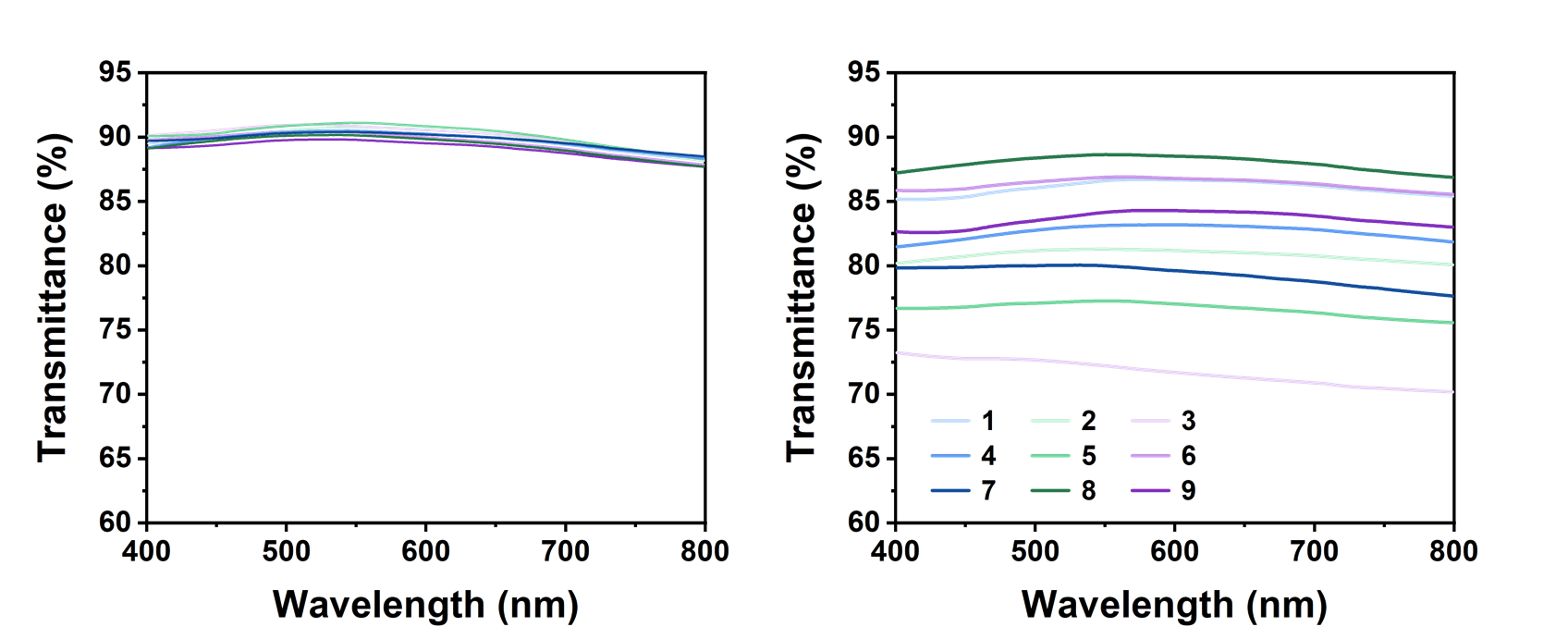


**Figure S2.** Light transmittance of 9 as-prepared samples before (left) and after (right) abrasion.


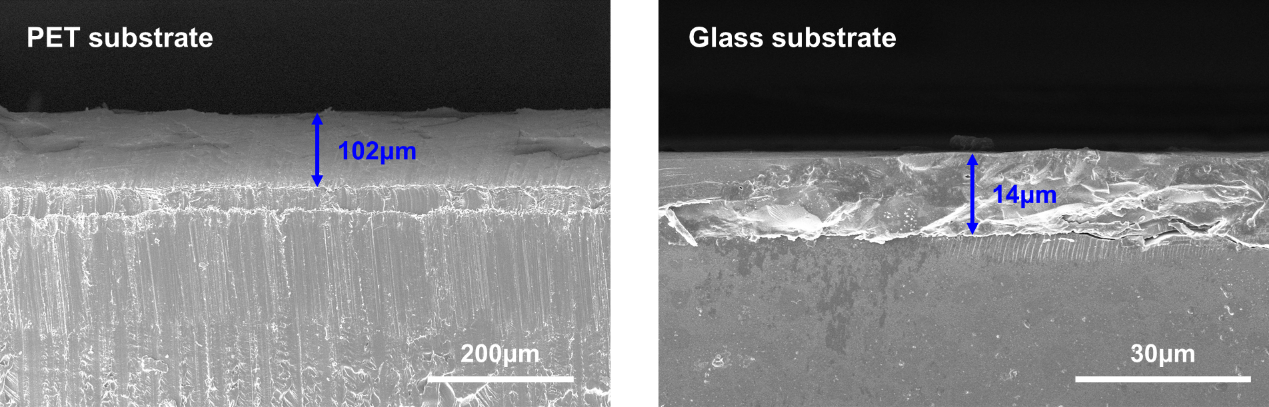


**Figure S3.** Thickness of the coating on different substrates.


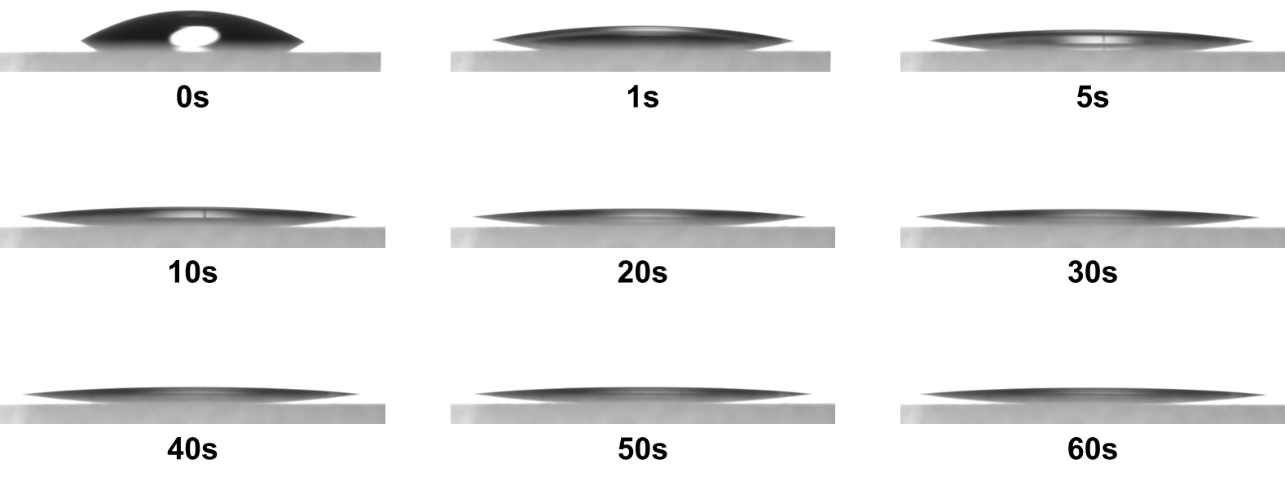


**Figure S4.** Variation of contact angle over time.


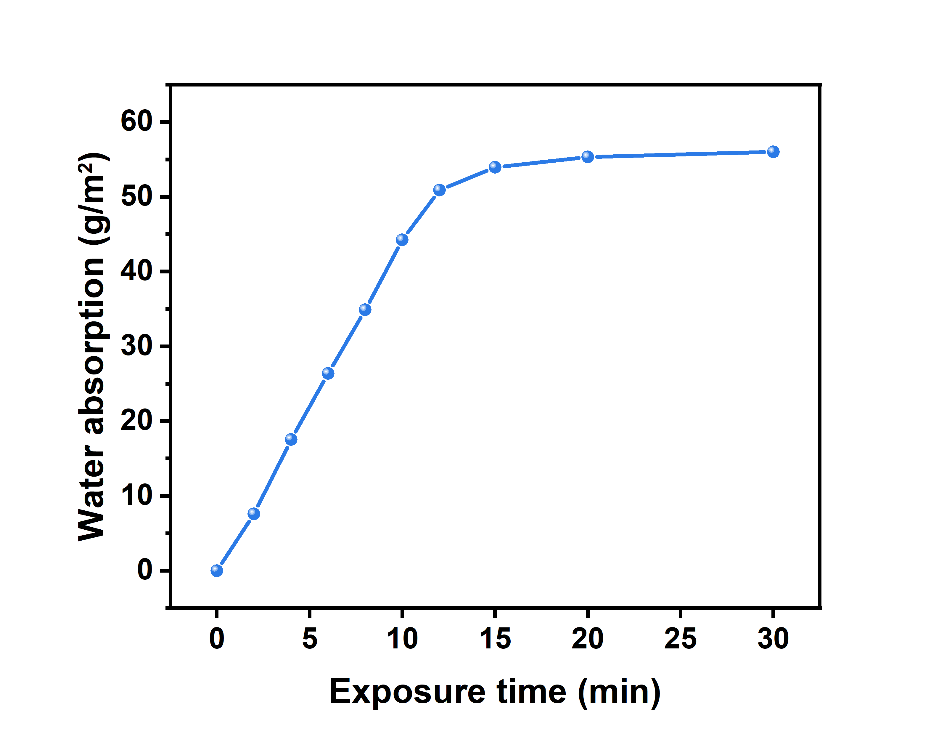


**Figure S5.** Water absorption of the coating over 30 min.


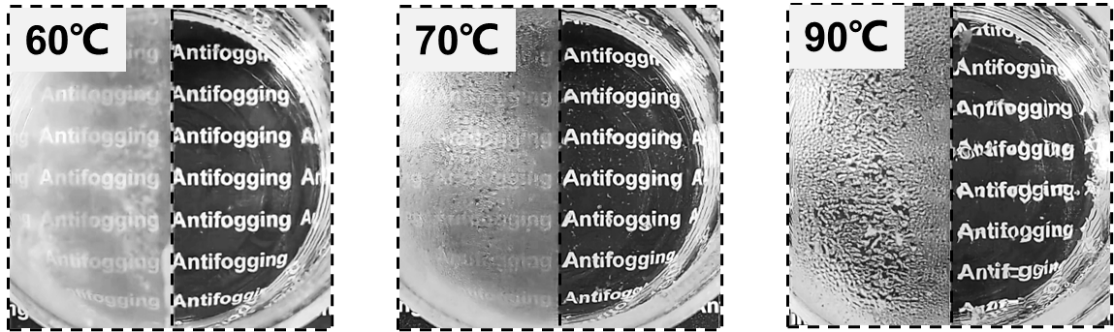


**Figure S6.** Antifogging performance (exposure to hot water 50 s) of the coating at different temperatures.


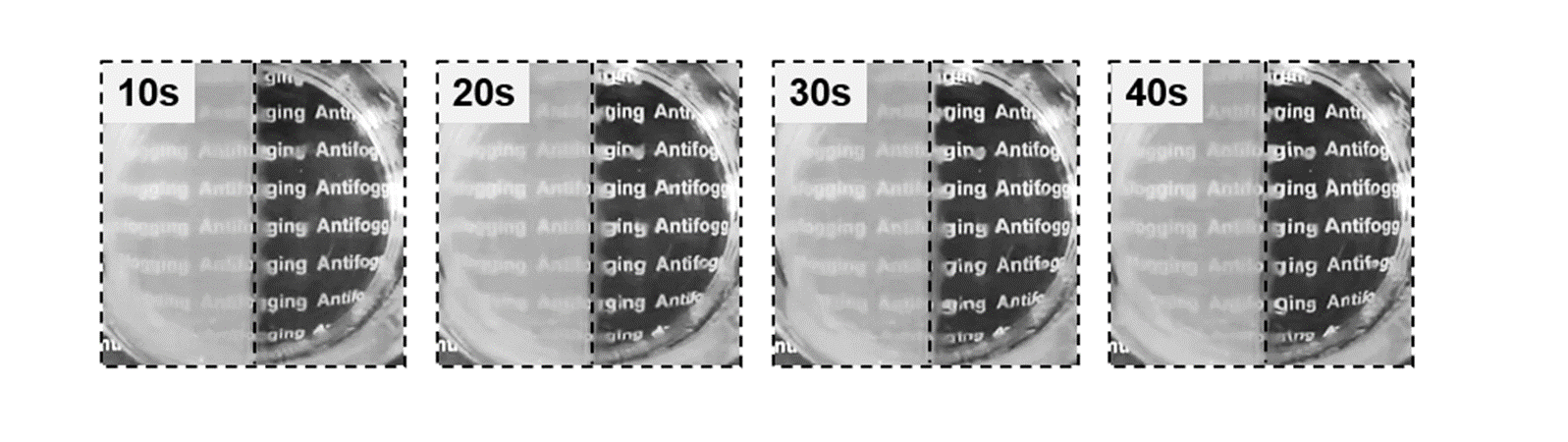


**Figure S7.** Antifogging performance (exposure to 80 °C hot water) of the coating on PET substrate.

**
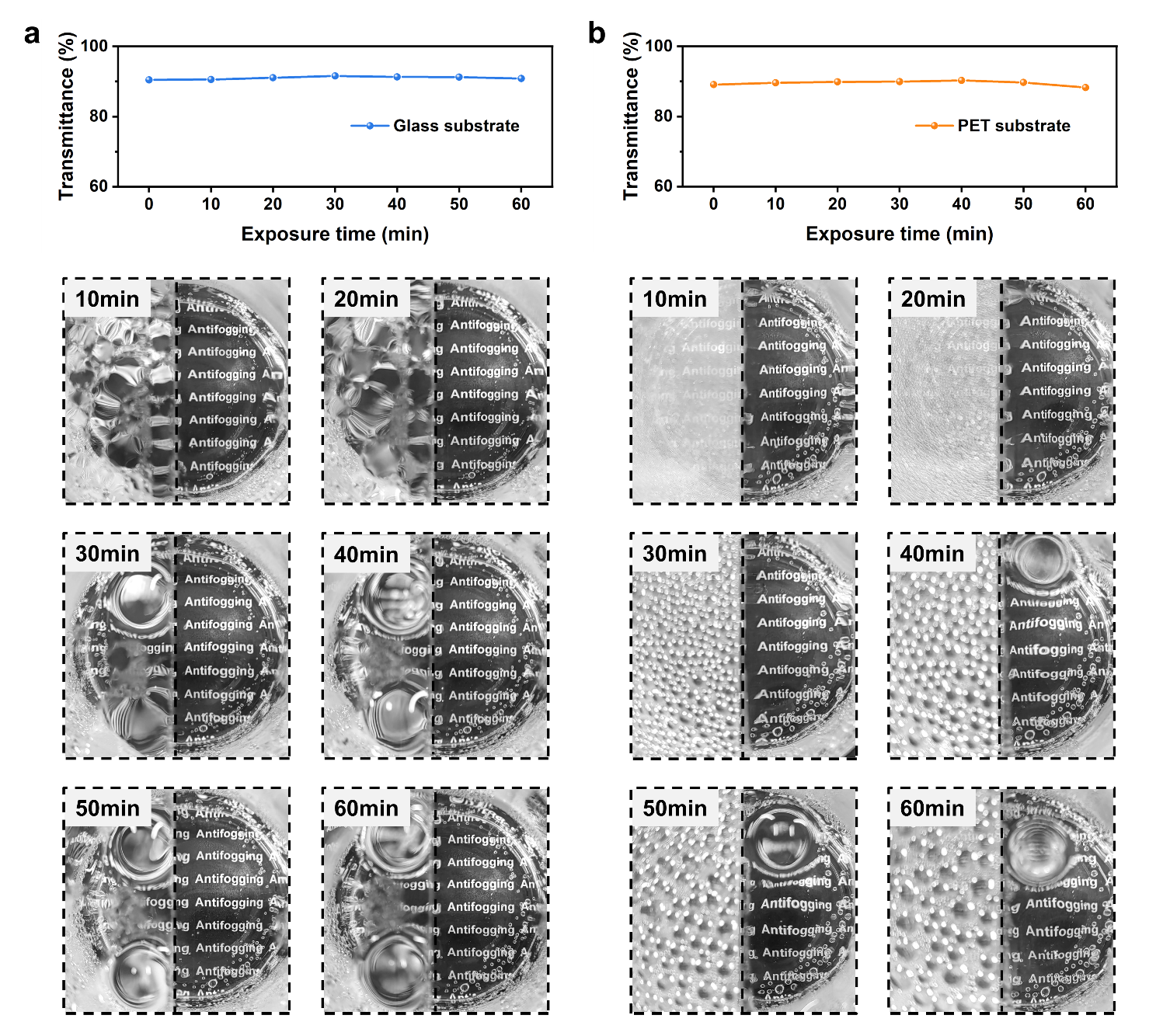
**

**Figure S8.** Long-lasting antifogging performance of the coating on a) glass substrate and b) PET substrate.

**
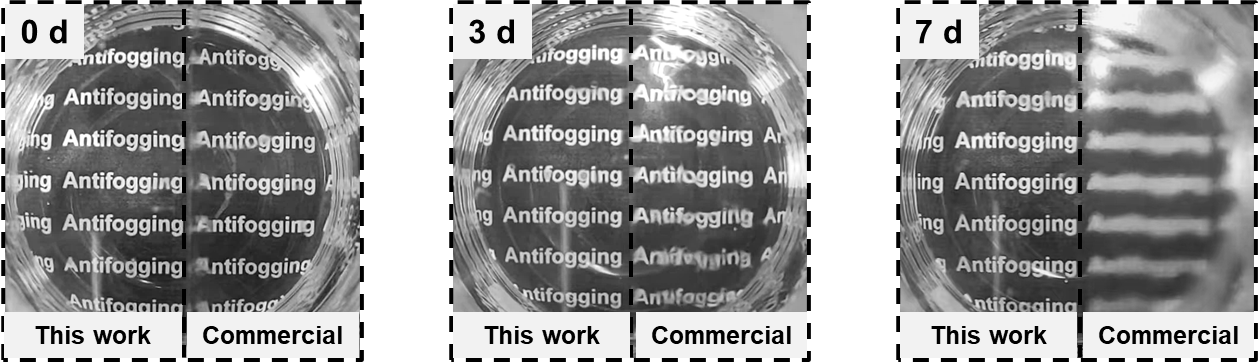
**

**Figure S9.** Antifogging performance of the PAA-ZCNF coating and commercial coating after storage for 0, 3, and 7 days.


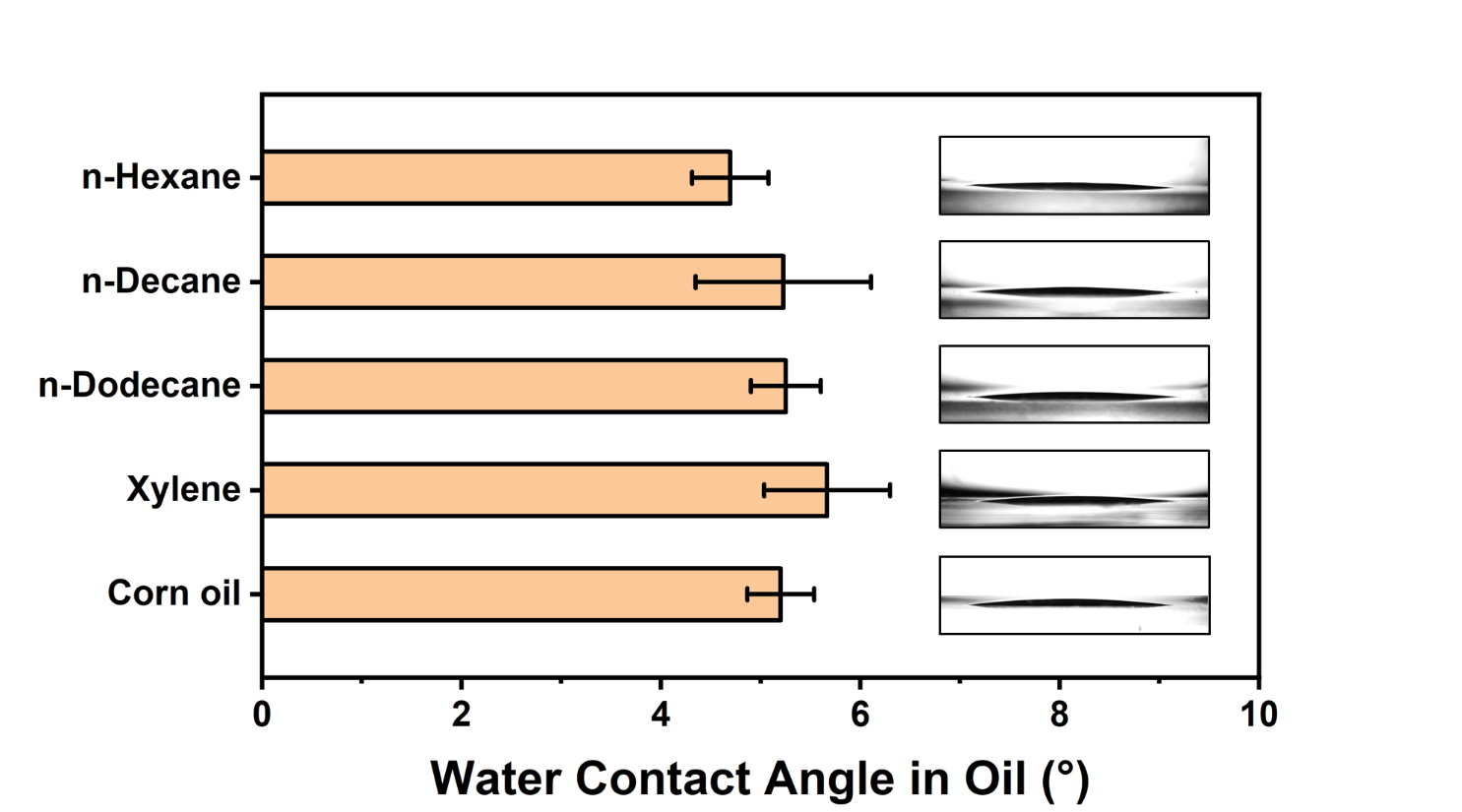


**Figure S10.** Water contact angle of the coating in different solvents.


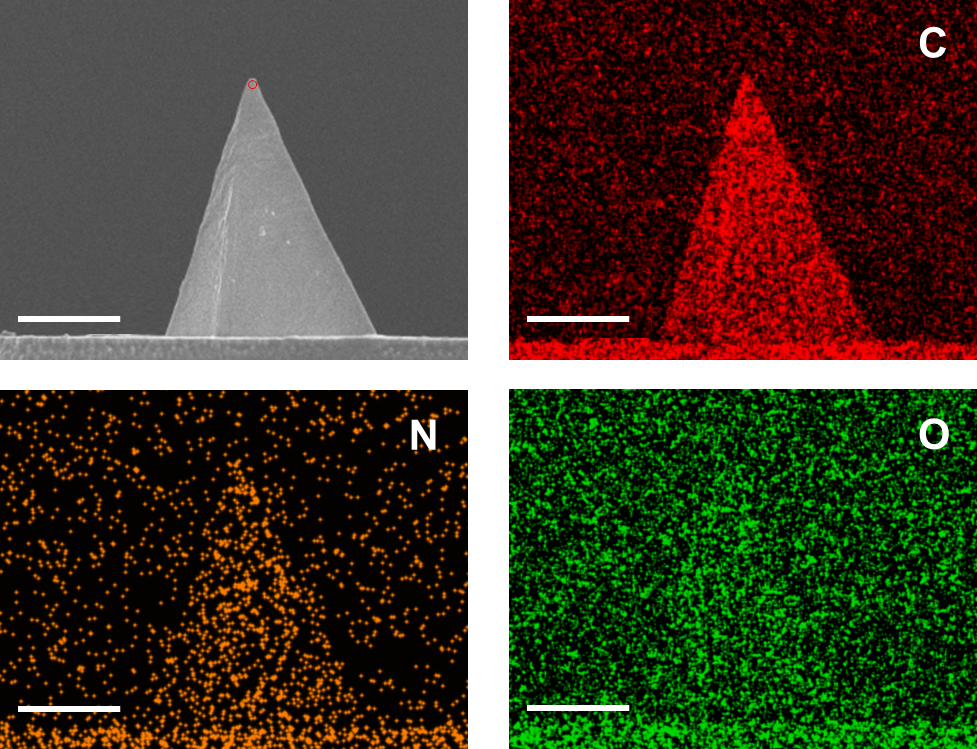


**Figure S11.** The elemental distribution map of the BSA-modified AFM probe. Scale bar = 2 μm.


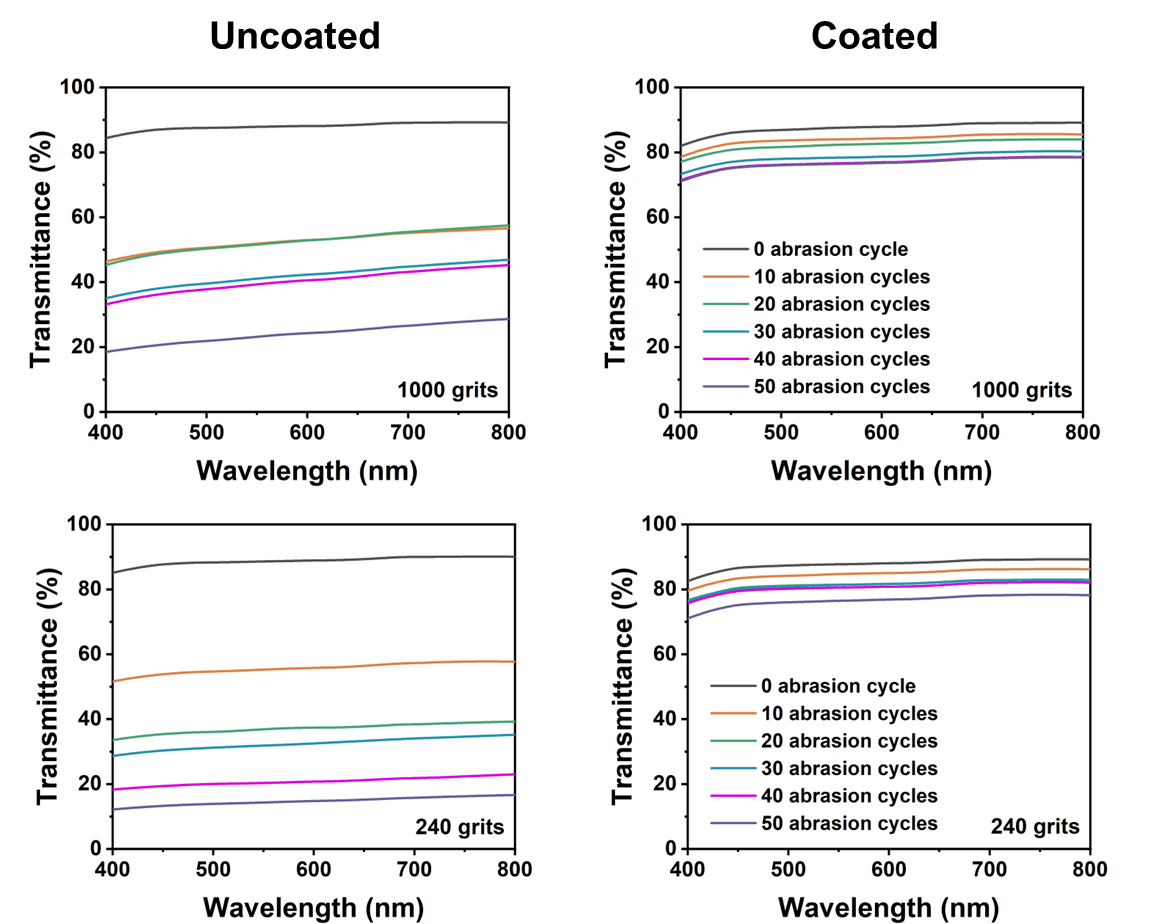


**Figure S12.** Comparison of light transmittance between coated and uncoated PET after abrasion.


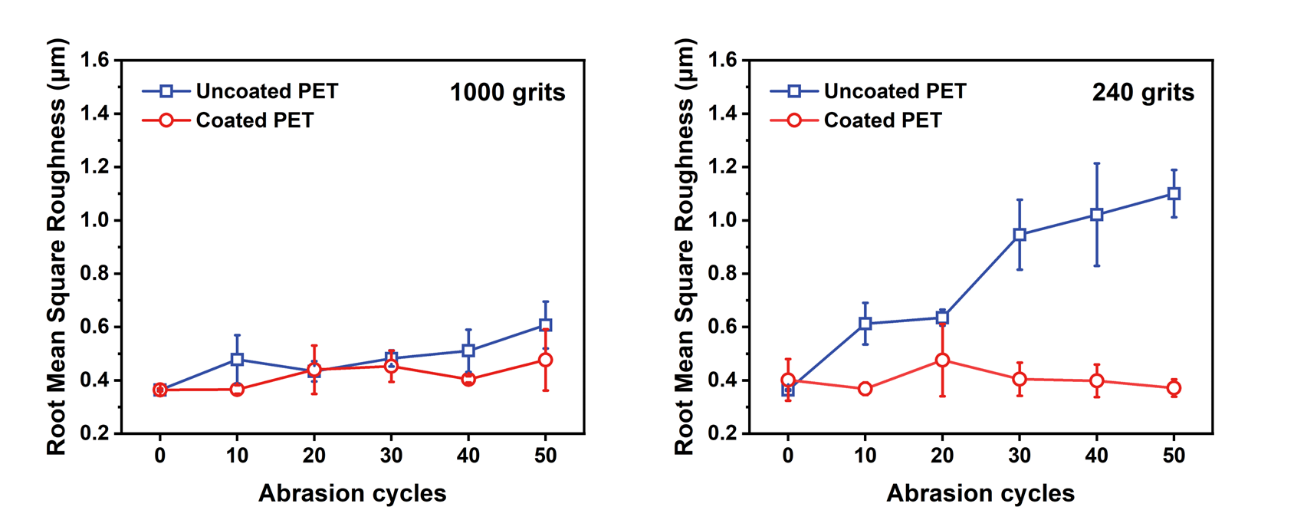


**Figure S13.** Comparison of root mean square roughness between coated and uncoated PET after abrasion.


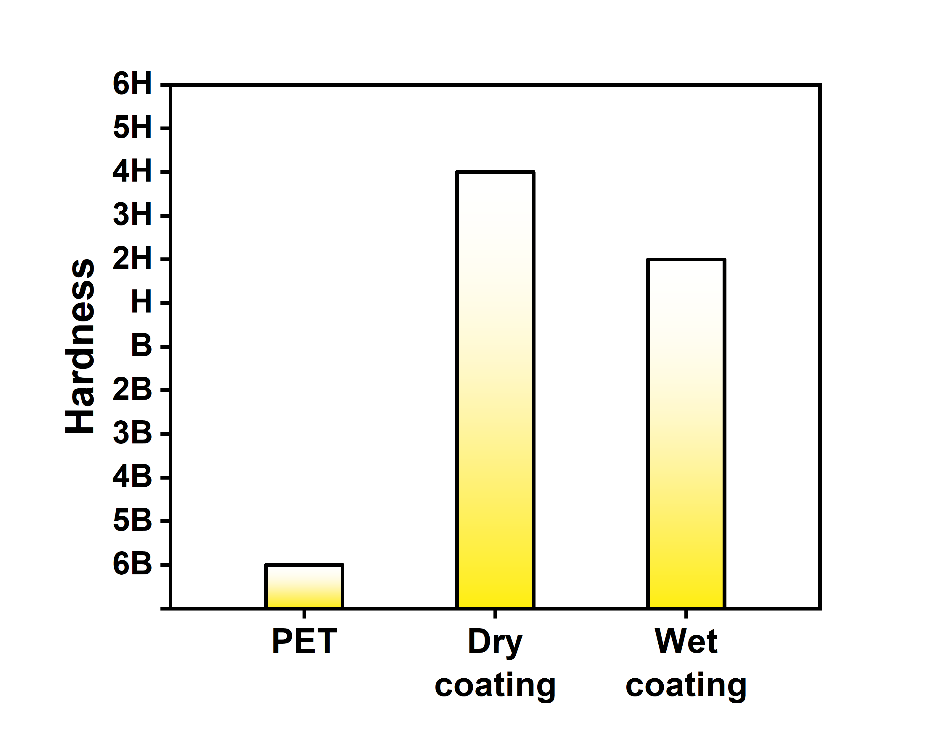


**Figure S14.** Pencil hardness of PET and coated PET.


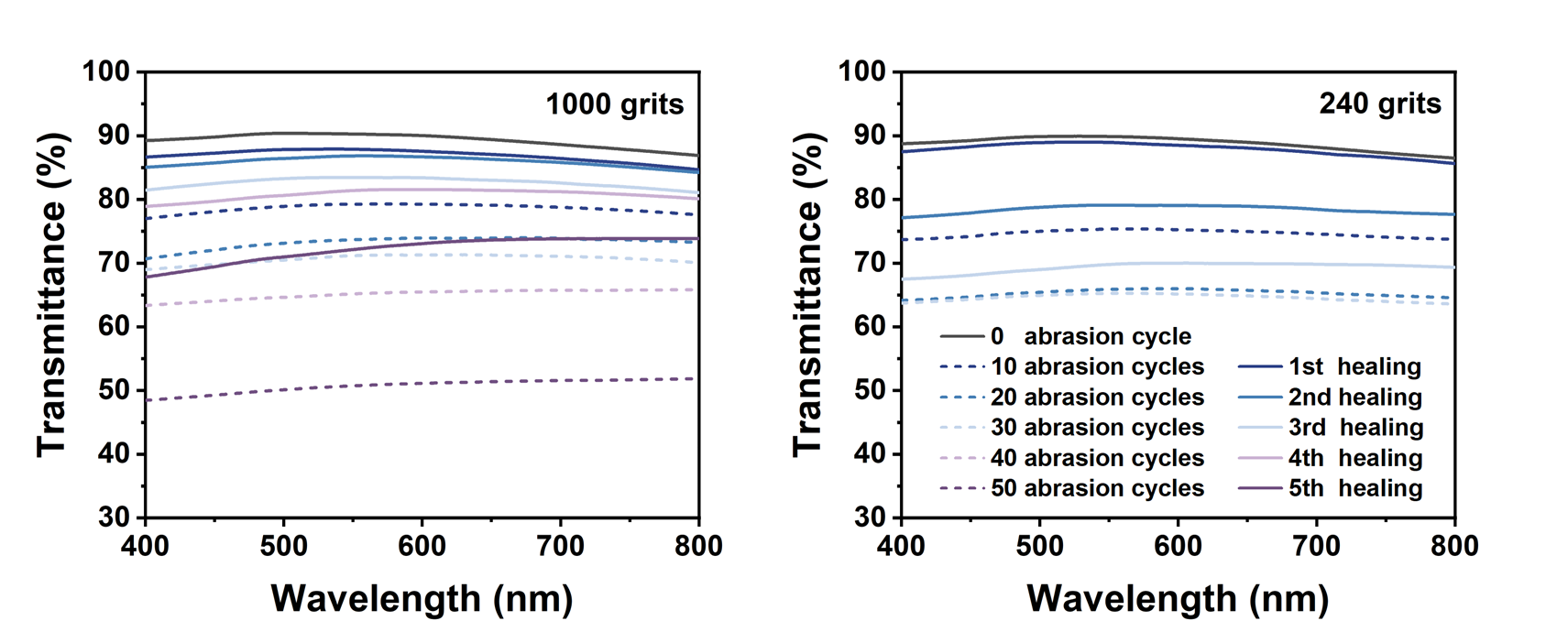


**Figure S15.** Light transmittance of the coating after multiple abrasion and healing cycles.


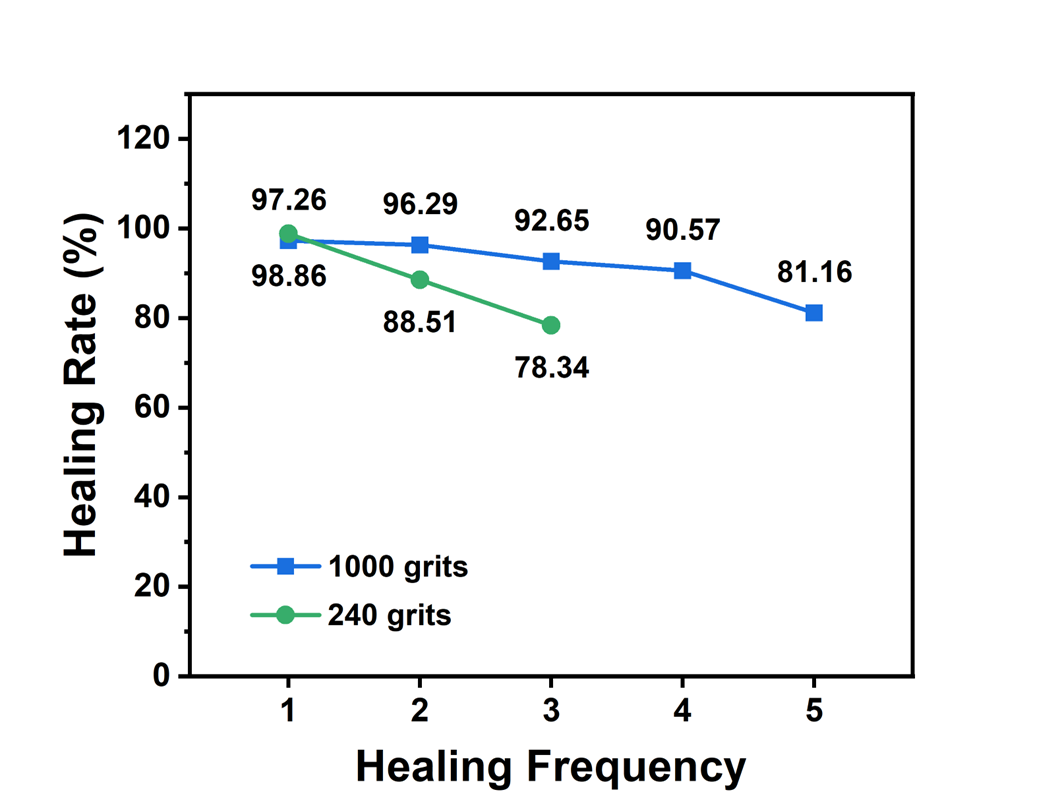


**Figure S16.** Healing rate of the coating.


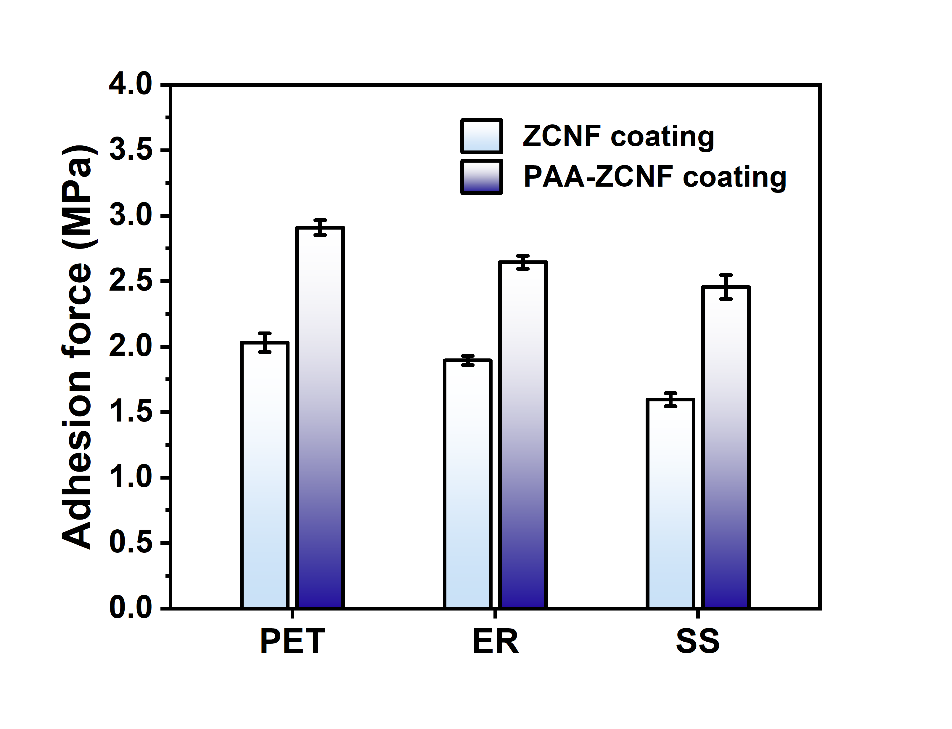


**Figure S17.** Adhesion force of the pure ZCNF coating and PAA-ZCNF coating on various substrates.


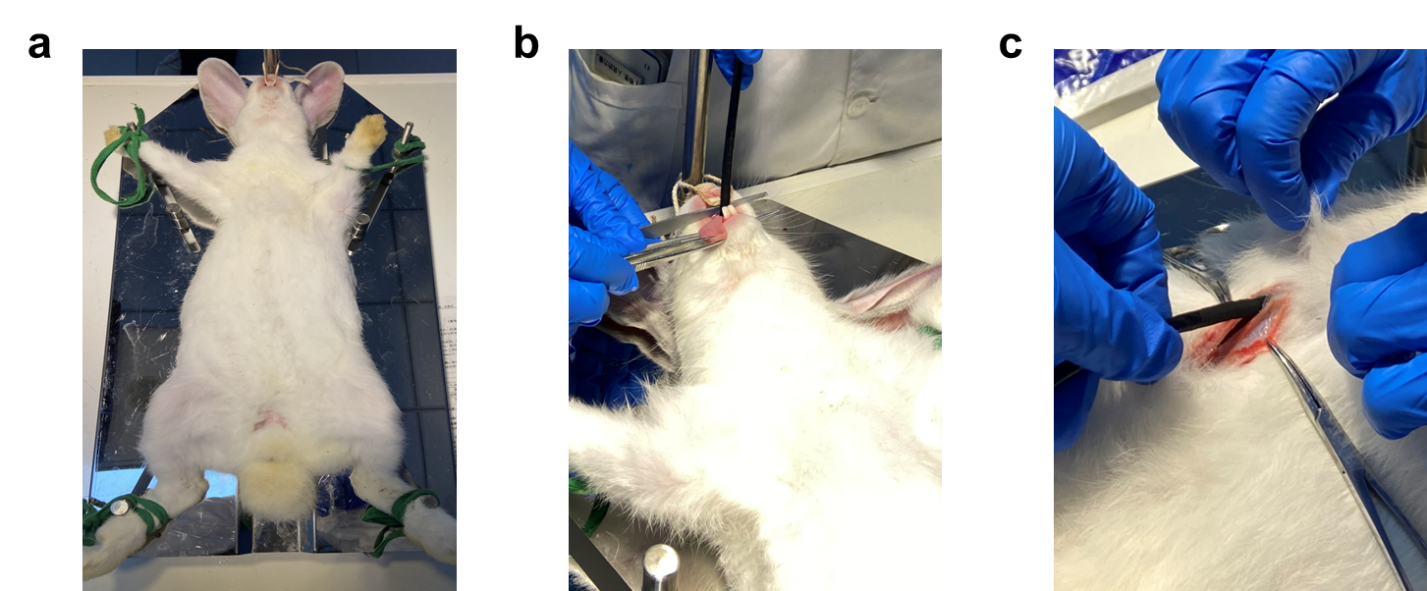


**Figure S18.** Photographs of the animal experimental procedure. a) The rabbit after anesthesia. b) Examination of the rabbit’s upper jaw. c) Examination of the rabbit’s abdominal cavity.


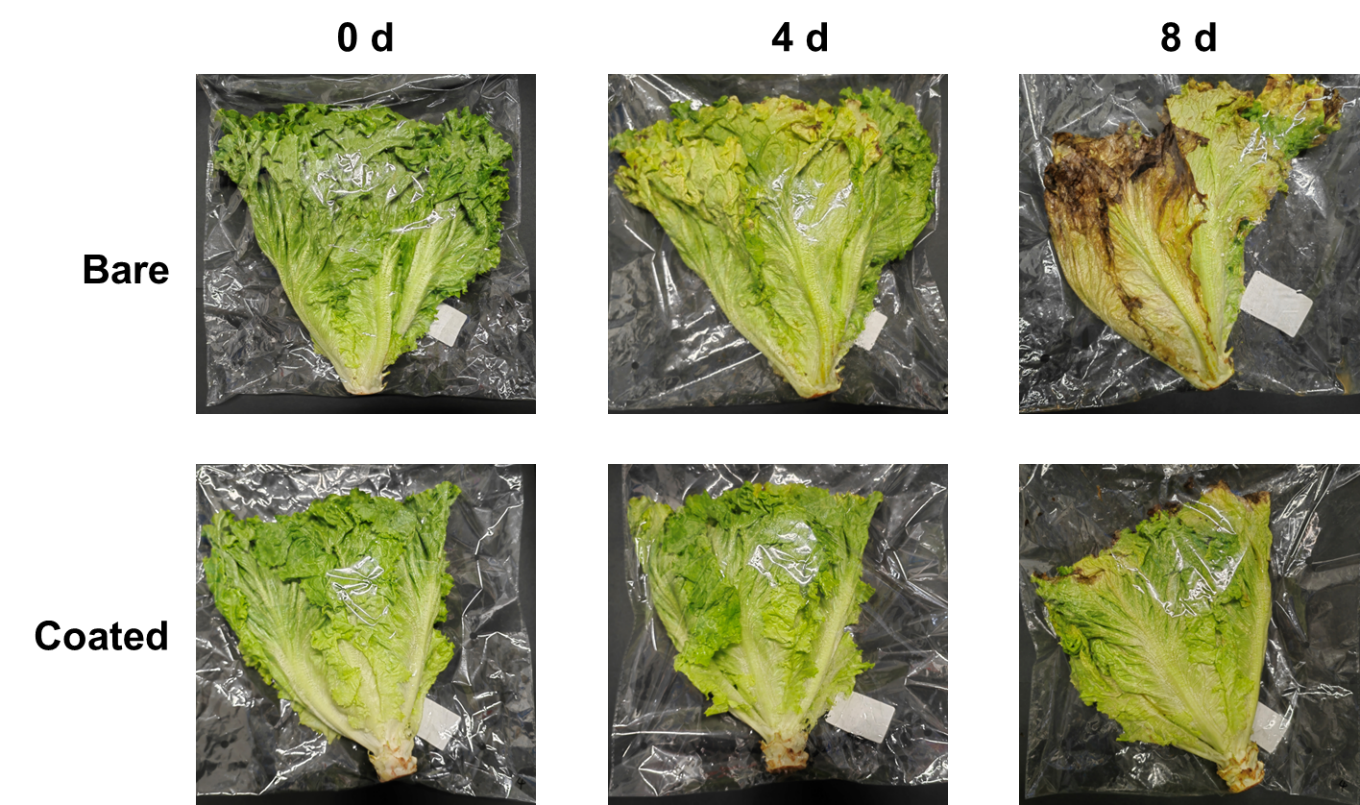


**Figure S19.** The lettuce in the packaging.


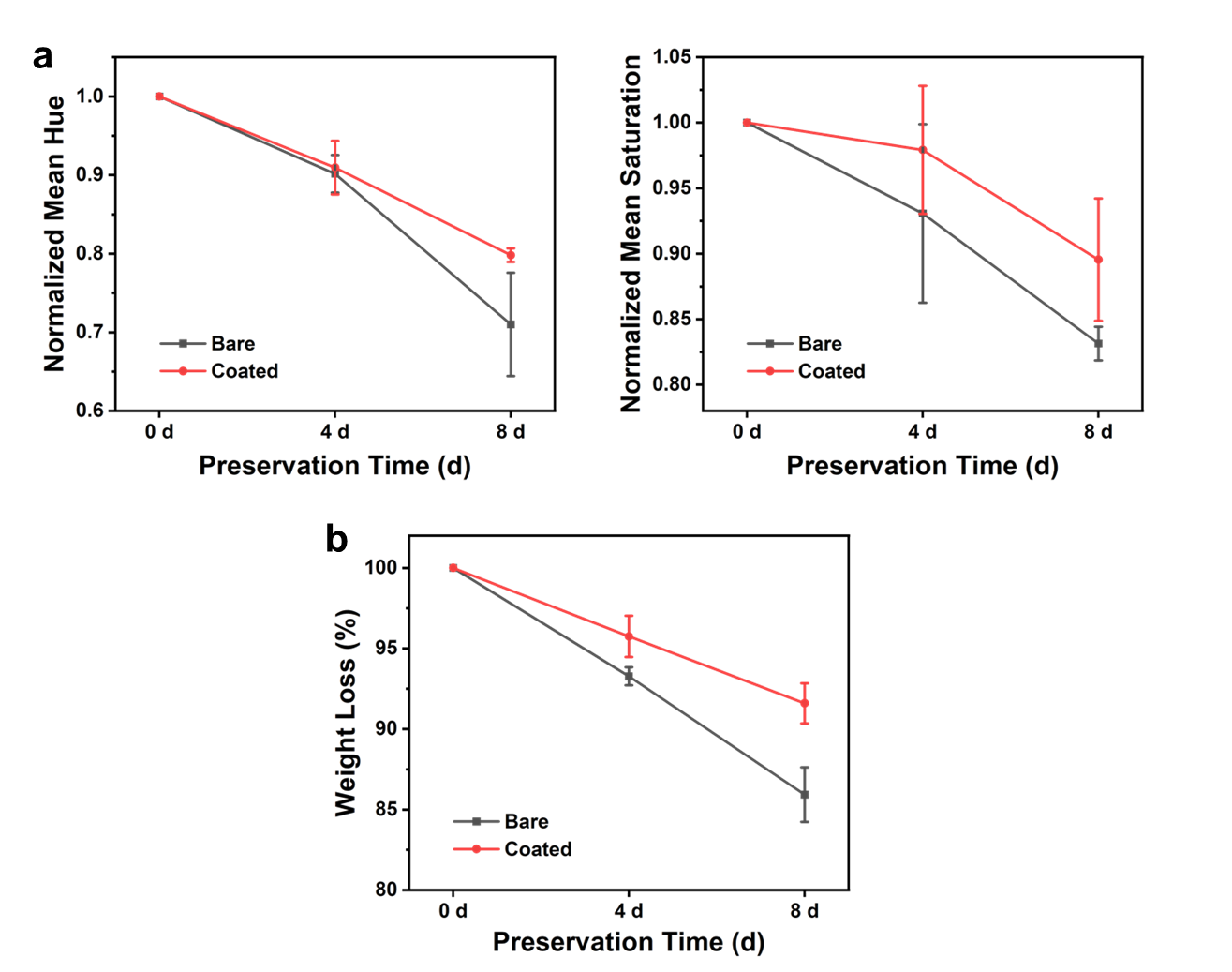


**Figure S20.** Changes of lettuce with preservation time. a) Hue and saturation changes in the lettuce photographs. b) Weight loss of lettuce.


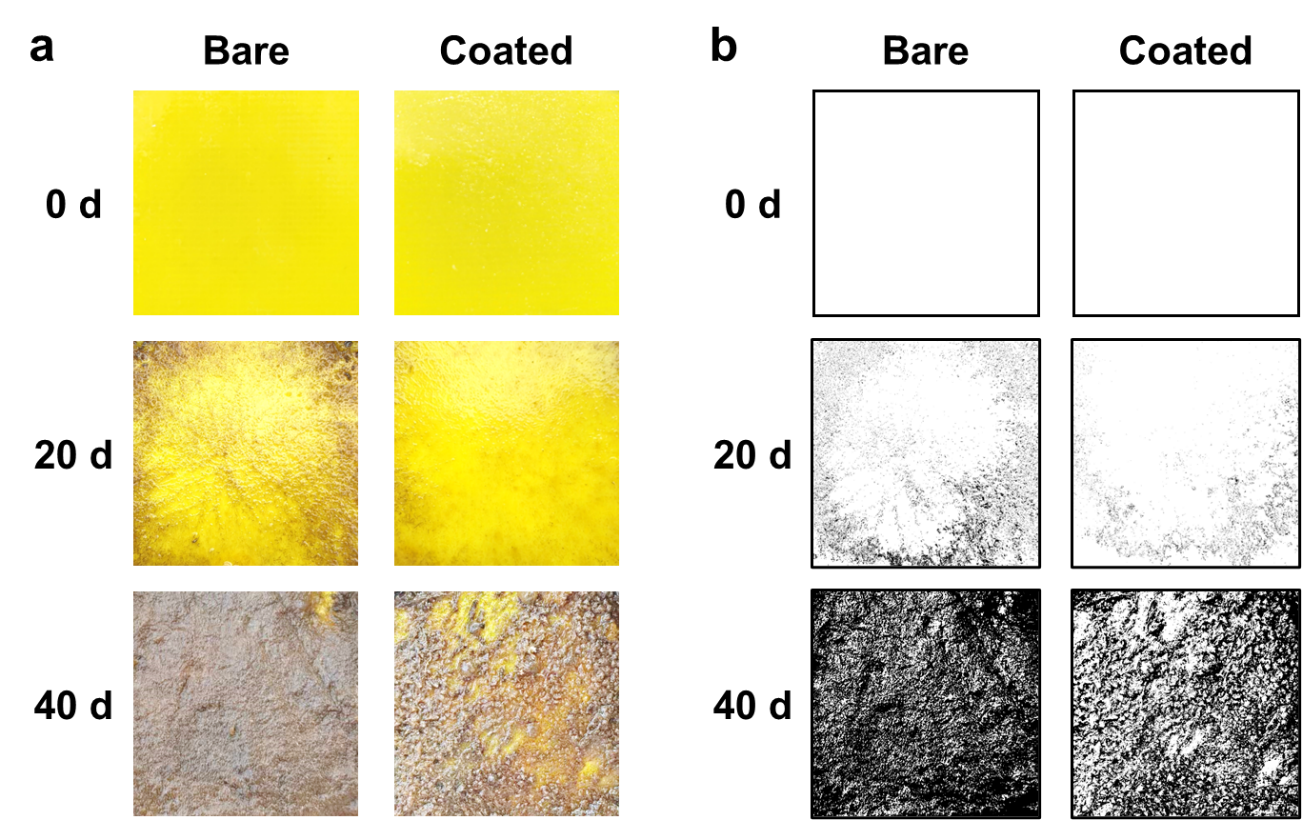


**Figure S21.** Marine field test. a) Photographs of the fouling on epoxy resin panels at different time points. b) Images processed using ImageJ software.


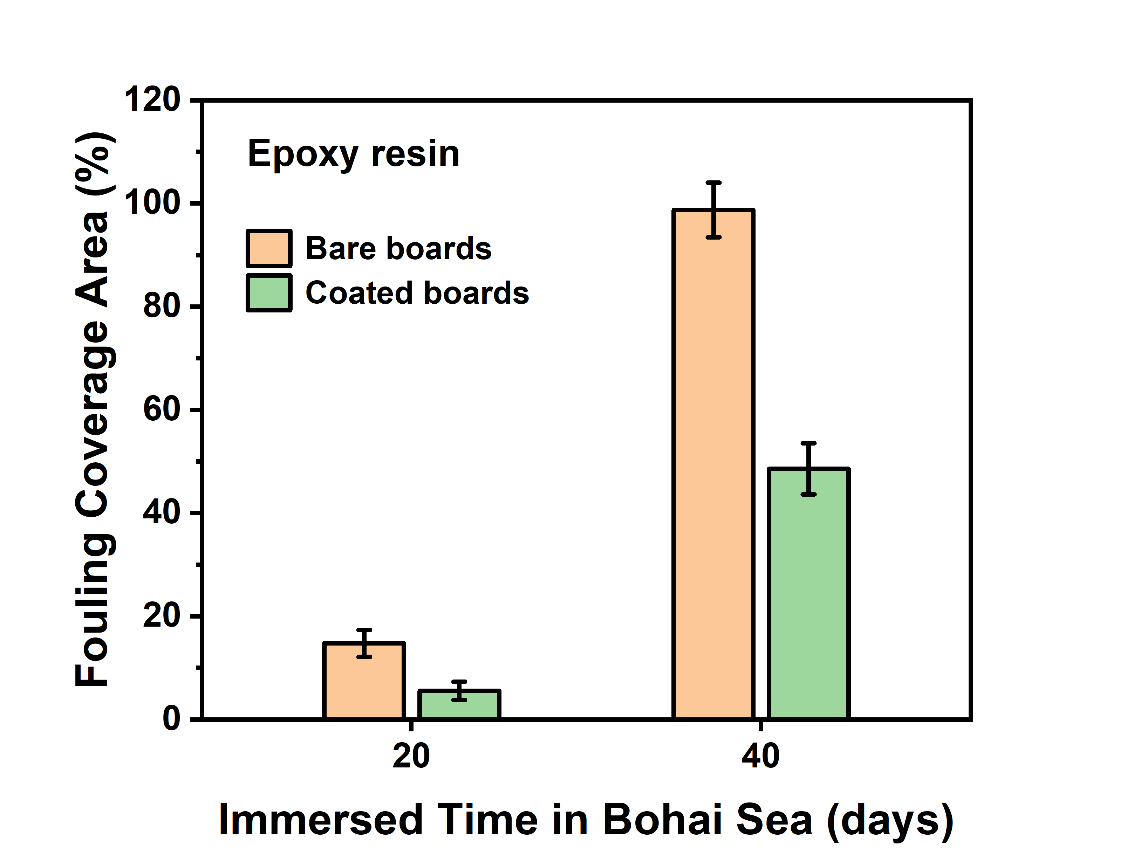


**Figure S22.** Statistical analysis of the fouling coverage area on panels at different time points in the marine field test.

**Reference**

[1] B. Y. Song, E. S. Zhang, Y. J. Shi, W. Wang, H. Zhu, S. J. Gallagher, S. Fischer, J. Rigney, E. Kim, Z. Q. Cao, *ACS Appl. Mater. Inter.* **2024**, *16*, 27908.

[2] M. X. Sun, S. H. Nie, Z. Wang, X. H. Jiang, *Chem Eng Sci* **2025**, *306*, 121218.

[3] F. Koschitzki, R. Wanka, L. Sobota, J. Koc, H. Gardner, K. Z. Hunsucker, G. W. Swain, A. Rosenhahn, *ACS Appl. Mater. Inter.* **2020**, *12*, 34148.

[4] Y. C. Zhang, J. S. Pan, Q. Y. Xie, C. F. Ma, G. Z. Zhang, *Adv Funct Mater* **2025**, DOI: 10.1002/adfm.202424994.

[5] H. Kim, Y. Song, S. Park, Y. Kim, H. Mun, J. Kim, S. Kim, K. G. Lee, S. G. Im, *Adv Funct Mater* **2022**, *32*, 2113253.

[6] M. B. Perez, D. A. Resendiz-Lara, Y. Matsushita, S. Kakinoki, Y. Iwasaki, M. A. Hempenius, S. de Beer, F. R. Wurm, *Adv Funct Mater* **2024**, *34*, 2316201.

[7] B. B. Berking, G. Poulladofonou, D. Karagrigoriou, D. A. Wilson, K. Neumann, *Angew Chem Int Edit* **2023**, *62*, e202308971.

[8] S. J. Wu, H. Yuk, J. J. Wu, C. S. Nabzdyk, X. H. Zhao, *Adv Mater* **2021**, *33*, 2007667.

[9] J. P. Hu, D. Z. Zhang, W. B. Li, Y. Li, G. R. Shan, M. Zuo, Y. H. Song, Z. L. Wu, L. Ma, Q. Zheng, M. Du, *ACS Appl. Mater. Inter.* **2024**, *16*, 6433.

[10] J. Akintola, Y. H. Chen, Z. A. Digby, J. B. Schlenoff, *ACS Appl. Mater. Inter.* **2023**, *15*, 50058.

[11] H. Y. Feng, J. B. Zhang, W. F. Yang, Y. F. Ma, R. Wang, S. H. Ma, M. R. Cai, B. Yu, F. Zhou, *ACS Appl. Mater. Inter.* **2021**, *13*, 50505.
